# Geographical distribution of *Amblyomma cajennense* (*sensu lato*) ticks (Parasitiformes: Ixodidae) in Brazil, with description of the nymph of *A. cajennense* (*sensu stricto*)

**DOI:** 10.1186/s13071-016-1460-2

**Published:** 2016-03-31

**Authors:** Thiago F. Martins, Amália R. M. Barbieri, Francisco B. Costa, Flávio A. Terassini, Luís M. A. Camargo, Cássio R. L. Peterka, Richard de C. Pacheco, Ricardo A. Dias, Pablo H. Nunes, Arlei Marcili, Alessandra Scofield, Artur K. Campos, Mauricio C. Horta, Aline G. A. Guilloux, Hector R. Benatti, Diego G. Ramirez, Darci M. Barros-Battesti, Marcelo B. Labruna

**Affiliations:** Departamento de Medicina Veterinária Preventiva e Saúde Animal, Faculdade de Medicina Veterinária e Zootecnia, Universidade de São Paulo, Av. Prof. Orlando Marques de Paiva 87, São Paulo, 05508-270 Brazil; Faculdade São Lucas, R. Alexandre Guimarães Areal 1927, Porto Velho, Rondônia 78916-450 Brazil; Departamento de Parasitologia, Instituto de Ciências Biomédicas, Universidade de São Paulo, Av. Professor Lineu Prestes 1374, São Paulo, 05389-970 Brazil; Coordenação Geral do Programa Nacional de Controle da Malária, Setor Comercial Sul, Quadra 04, Bloco A, Edifício Principal, 6° Andar, Brasília, Distrito Federal 70304-000 Brazil; Departamento de Ciências Básicas e Produção Animal, Faculdade de Agronomia, Medicina Veterinária e Zootecnia, Universidade Federal de Mato Grosso, Av. Fernando Corrêa da Costa 2367, Cuiabá, Mato Grosso 78060-900 Brazil; Instituto Latino-Americano de Ciências da Vida e da Natureza, Universidade Federal da Integração Latino-Americana, Av. Tarquínio Joslin dos Santos 1000, Foz do Iguaçu, Paraná 85870-901 Brazil; Universidade de Santo Amaro, R. Prof. Enéas de Siqueira Neto 340, São Paulo, 04829-300 Brazil; Curso de Medicina Veterinária, Faculdade de Medicina Veterinária, Universidade Federal do Pará, Av. Maximino Porpino 1000, Castanhal, Pará 68740-000 Brazil; Deparatamento de Veterinária, Universidade Federal de Viçosa, Av. PH Rolfs, s/n, Viçosa, Minas Gerais 36570-000 Brazil; Universidade Federal do Vale do São Francisco, Campus de Ciências Agrárias, Rodovia BR 407, Km 12, Lote 543 - Projeto de Irrigação Senador Nilo Coelho, s/n, Petrolina, Pernambuco 56300-990 Brazil; Laboratório Especial de Coleções Zoológicas, Instituto Butantan, Av. Vital Brasil 1500, São Paulo, 05503-900 Brazil

**Keywords:** *Amblyomma cajennense*, *Amblyomma sculptum*, Distribution, ITS2, Nymph

## Abstract

**Background:**

Until recently, *Amblyomma cajennense* (Fabricius, 1787) was considered to represent a single tick species in the New World. Recent studies have split this taxon into six species. While the *A. cajennense* species complex or *A. cajennense* (*sensu lato*) (*s.l.*) is currently represented by two species in Brazil, *A. cajennense* (*sensu stricto*) (*s.s.*) and *Amblyomma sculptum* Berlese, 1888*,* their geographical distribution is poorly known*.*

**Methods:**

The distribution of the *A. cajennense* (*s.l.*) in Brazil was determined by morphological examination of all lots of *A. cajennense* (*s.l.*) in two large tick collections of Brazil, and by collecting new material during three field expeditions in the possible transition areas between the distribution ranges of *A. cajennense* (*s.s.*) and *A. sculptum.* Phylogenetic analysis inferred from the ITS2 rRNA gene was used to validate morphological results. Morphological description of the nymphal stage of *A. cajennense* (*s.s.*) is provided based on laboratory-reared specimens.

**Results:**

From the tick collections, a total 12,512 adult ticks were examined and identified as 312 *A. cajennense* (*s.s.*), 6,252 *A. sculptum* and 5,948 *A. cajennense* (*s.l.*). A total of 1,746 ticks from 77 localities were collected during field expeditions, and were identified as 249 *A. cajennense* (*s.s.*), 443 *A. sculptum,* and 1,054 *A. cajennense* (*s.l.*) [these *A. cajennense* (*s.l.*) ticks were considered to be males of either *A. cajennense* (*s.s.*) or *A. sculptum*]. At least 23 localities contained the presence of both *A. cajennense* (*s.s.*) and *A. sculptum* in sympatry. DNA sequences of the ITS2 gene of 50 ticks from 30 localities confirmed the results of the morphological analyses. The nymph of *A. cajennense* (*s.s.*) is morphologically very similar to *A. sculptum.*

**Conclusion:**

Our results confirmed that *A. cajennense* (*s.l.*) is currently represented in Brazil by only two species, *A. cajennense* (*s.s.*) and *A. sculptum.* While these species have distinct distribution areas in the country, they are found in sympatry in some transition areas. The current distribution of *A. cajennense* (*s.l.*) has important implications to public health, since in Brazil *A. sculptum* is the most important vector of the bacterium *Rickettsia rickettsii*, the etiological agent of Brazilian spotted fever.

**Electronic supplementary material:**

The online version of this article (doi:10.1186/s13071-016-1460-2) contains supplementary material, which is available to authorized users.

## Background

Until recently, the taxon *Amblyomma cajennense* (Fabricius, 1787) was considered to represent a single tick species occurring in southern United States, Mexico, Central America, Caribbean and all countries of South America with the exception of Chile and Uruguay [1]. A recent morphological study [2], supported by biological [3, 4] and molecular analyses [5], split this taxon into six valid species, namely *A. cajennense sensu stricto* (*s.s*.) (restricted to the Amazonian region), *Amblyomma mixtum* Koch, 1844 (from Texas to western Ecuador), *Amblyomma sculptum* Berlese, 1888 (northern Argentina, Bolivia, Paraguay, Brazil), *Amblyomma interandinum* Beati, Nava & Cáceres, 2014 (inter-Andean valley of Peru), *Amblyomma tonelliae* Nava, Beati & Labruna, 2014 (dry areas of northern Argentina, Bolivia and Paraguay), and *Amblyomma patinoi* Labruna, Nava & Beati, 2014 (Eastern Andes of Colombia) [2, 5]. With this new classification, the *A. cajennense* species complex or *A. cajennense* (*sensu lato*) (*s.l.*) is currently represented by two species in Brazil, *A. cajennense* (*sensu stricto*) and *A. sculptum*, which can be morphologically separated only by examination of the genital aperture of females [2]. However, as stated by Nava *et al.* [2], the known geographical distribution of these species is still incomplete. In addition, it is not known if there is any geographical overlap between *A. cajennense* (*s.s.*) and *A. sculptum*.

There have been four previous descriptions of nymphs of *A. cajennense* (*s.l.*): two of *A. cajennense* (*s.l.*) [6, 7], one of *A. sculptum* [8], and one of *A. tonelliae* [8]. Indeed, the description of the nymph of *A. cajennense* by Martins *et al.* [7] refers to *A. sculptum*, since the described ticks were from the state of São Paulo, southeastern Brazil, a typical area for the occurrence of *A. sculptum* [2]. While Cooley & Kohls [6] did not mention the specific origin of the described nymphs, it is possible that their nymphs were *A. mixtum*, the only species of *A. cajennense* (*s.l.*) known to occur in North America [2]*.*

The present study aimed to determine the geographical distribution of *A. cajennense* (*s.l.*) in Brazil. For this purpose, all lots of *A. cajennense* (*s.l.*) in two large tick collections of Brazil were revised. New material was acquired during three field expeditions in the possible transition areas between the distribution ranges of *A. cajennense* (*s.s.*) and *A. sculptum*, where these two species might overlap*.* In addition, molecular analyses were performed on representative tick specimens from different regions, in order to confirm their taxonomic identification. Finally, a morphological description of the nymphal stage of *A. cajennense* (*s.s.*) is provided for the first time.

## Methods

### Examination of ticks deposited at tick collections

**Fig. 1:**
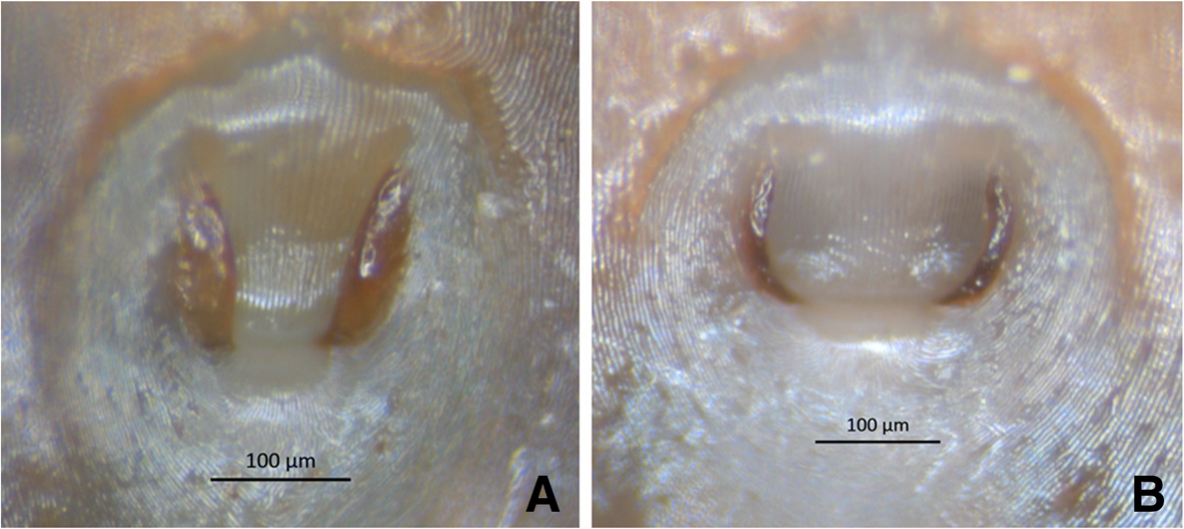
Genital aperture of adult females. **a** “V” shaped in *Amblyomma cajennense* (*sensu stricto*), a specimen from Governador Jorge Teixeira, Rondônia, Brazil; **b** “U” shaped in *Amblyomma sculptum*, a specimen from Pirassununga, São Paulo, Brazil

The following tick collections were assessed: the “Acari Colletion of the Instituto Butantan” (IBSP), São Paulo, Brazil; and the “Coleção Nacional de Carrapatos” (CNC) of the University of São Paulo, São Paulo, Brazil. In each tick collection, all lots containing adults of *A. cajennense* (*s.l.*) were examined morphologically under a stereomicroscope. Females were identified as *A. cajennense* (*s.s.*) or *A. sculptum,* based on the genital aperture morphology (Fig. 1), i.e. V-shaped in the former and U-shaped in the latter [2]. Due to the lack of discriminating characters to separate males of *A. cajennense* (*s.s.*) and *A. sculptum* (see Nava *et al*. [2]), males were morphologically identified as *A. cajennense* (*s.l.*) From each lot, the geographical coordinates of the municipality was retrieved from the “Instituto Brasileiro de Geografia e Estatística” (IBGE) website (www.ibge.gov.br).

### Field expeditions for collection of ticks

Three field expeditions were performed in the possible transition areas of the distribution ranges of *A. cajennense* (*s.s.*) and *A. sculptum* in Brazil. One expedition, during January 2012, included areas of the states of Maranhão, Pará, Tocantins, and Goiás. These states were considered to represent the eastern transition area of the two species. A second expedition, also during January 2012, included areas of the states of Rondônia and Mato Grosso, which were considered to represent the western transition area of two species in Brazil. Finally, a third expedition, during February 2013, included the state of Mato Grosso, in areas that could represent the central transition area of the two species in Brazil. The three expeditions were carried out during January or February because previous studies indicated that these months correspond in Brazil to the highest abundance of the adult stage of *A. cajennense* (*s.s.*) [9] or *A. sculptum* [10]. Each expedition consisted of driving a car through regular roads and stopping at regular distances (usually every ≈ 50 Km) for examination of horses and pigs (main domestic hosts for *A. cajennense* (*s.l.*) in Brazil [11, 12]) for tick infestations, and/or for collecting questing ticks on the vegetation by dragging and visual search methods [13]. In addition, road-killed tapirs (*Tapirus terrestris* Linnaeus, 1758) and anteaters (*Myrmecophaga tridactyla* Linnaeus, 1758), two main hosts for *A. cajennense* (*s.l.*) [11, 12] were occasionally examined for tick infestations. Every time *A. cajennense* (*s.l.*) ticks were found, they were collected in plastic vials containing absolute ethanol, and the geographic coordinates were recorded with the use of a Global Position System apparatus (GPS Garmin, model GPS V, Olathe, KA, USA). All ticks were taken to the laboratory for taxonomic identification as stated above.

### Molecular analyses

For molecular analyses, we used one to three specimens from each given location. From the material collected in the field expeditions, we prioritised male specimens from locations where no female specimen was available, since species identification through morphological examination can be achieved only for females. In addition, we processed male and female specimens taken from the CNC tick collection, from different locations of Brazil, in order to have representative specimens from nearly all Brazilian states where *A. cajennense* (*s.l.*) has been reported.

Each tick specimen was individually submitted to DNA extraction by the guanidine thiocyanate method [14], and tested by a PCR assay targeting the entire tick ITS2 rRNA gene, with primers corresponding to the flanking regions (5.8S rRNA 3′-end, and 28S rRNA 5′-end), as previously described [15]. All PCR products of the expected size (≈1,100 bp) were purified with ExoSap (USB, Cleveland, OH, USA) and sequenced in an ABI automated sequencer (Applied Biosystems/Thermo Fisher Scientific, model ABI 3500 Genetic Analyser, Foster City, CA, USA) with the same primers used for PCR.

### Phylogenetic analysis

The ITS2 sequences generated in the present study, excluding the rRNA 5.8S and 28S flanking regions, were aligned with GenBank sequences for *A. sculptum* from Minas Gerais, Brazil (JN866842, JN866843), São Paulo, Brazil (JN866846), and Argentina (JN866835); *A. mixtum* from Costa Rica (JN866853) and Mexico (JN866886); *A. patinoi* from Colombia (JN866881, JN866882); *A. tonelliae* from Argentina (JN866895, JN866896, JN866897); *A. interandinum* from Peru (JN866900, JN866902, JN866905); and *A. cajennense* (*s.s.*) from French Guiana (JN866864) [2, 5] using Clustal X [16], and were manually adjusted using GeneDoc, version 2.6.01 [17]. Thereafter, the ITS2 sequences of *Amblyomma aureolatum* (Pallas, 1772) (AF469611) and *Amblyomma americanum* (Linnaeus, 1758) (AF291874) were added as outgroup.

The ITS2 alignment was used to construct a phylogenetic tree using maximum parsimony, as implemented in PAUP version 4.0b10 [18] with 500 bootstrap replicates, random stepwise addition starting trees (with random addition sequences) and TBR branch swapping. Bayesian analysis was performed using MrBayes v3.1.2 [19] with four independent Markov chain runs for 5,000,000 metropolis-coupled MCMC generations, sampling a tree every 100^th^ generation. The first 25 % of the trees represented “burn-in”, and the remaining trees were used to calculate Bayesian posterior probabilities, which are presented in the consensus tree.

### Distribution maps

For construction of distribution maps of *A. cajennense* (*s.s.*) and *A. sculptum* in Brazil, we used the geographical coordinates of all lots of these two species in the IBSP and CNC tick collections, plus the coordinates of the ticks collected during the three field expeditions of the present study. Tick locations were plotted on four different maps: (i) geopolitical, (ii) biomes, (iii) climate, and (iv) remaining natural vegetation cover (forest remains). Map sources used in the present study were obtained from the “Instituto Brasileiro de Geografia e Estatística” (IBGE) website (www.ibge.gov.br) and the “Ministério do Meio Ambiente”(MMA) website (www.mma.gov.br). The distribution maps were constructed with the use of ArcGIS version 10.0 (ESRI).

### Description of the nymph of *A. cajennense* (*s.s.*)

In July 2014, a tick colony of *A. cajennense* (*s.s.*) was established in the laboratory, started with adult ticks collected on the vegetation at Governador Jorge Teixeira municipality (10°31′S, 62°38′W), state of Rondônia, Brazil. Unfed F_1_ nymphs, 15–30 day-old, were killed in hot water (70–80 °C) and immediately preserved in 70 % alcohol until further processing for description. Nymphal description, based on optical microscopy, followed Martins et al. [7]. Measurements were taken from 10 specimens using the programme Image-Pro Plus 5.1 for analysis of images and morphometry, fitted to an Olympus SZX stereoscope microscope. All measurements are in micrometres and are given as the range followed by the mean in parentheses. These nymphs were deposited in the CNC (accession number CNC-2913). Nymphs were prepared for scanning electron microscopy (SEM) following previously described techniques [20] and SEM micrographs were taken using a HITACHI TM3000 Scanning Electron Microscope.

### Ethical approval

This work has been approved by the Ethic Committee in the Use of Animals of the Faculty of Veterinary Medicine of the University of Sao Paulo (project number 2660/2012).

## Results

### Examination of ticks deposited at tick collections

A total of 335 lots of adult ticks *A. cajennense* (*s.l.*) were examined from the IBSP tick collection. These were identified as 67 females of *A. cajennense* (*s.s.*), 648 females of *A. sculptum*, and 978 males of *A. cajennense* (*s.l.*) Similarly, a total of 545 lots of adult ticks *A. cajennense* (*s.l.*) were examined at the CNC tick collection. These were identified as 245 females of *A. cajennense* (*s.s.*), 5,604 females of *A. sculptum* and 4,970 males of *A. cajennense* (*s.l.*) For each lot examined, host, date, and locality data (including geographical coordinates) are listed in Additional file 1: Table S1).

From a total of 55 lots of *A. cajennense* (*s.s.*) with host data (total: at least 16 different host species), the most common hosts were *Equus caballus* Linnaeus, 1758 (12 lots, 21.8 %) and *T. terrestris* (10 lots, 18.2 %), followed by *Canis lupus familiaris* Linnaeus, 1758 (6 lots, 10.9 %), *Bos taurus* Linnaeus, 1758 (5 lots, 9.1 %), *M. tridactyla* (4 lots, 7.3 %), and *Homo sapiens* Linnaeus, 1758 (3 lots, 5.5 %). From a total of 438 lots of *A. sculptum* with host data (total: at least 48 different host species)*,* the most common hosts were *H. sapiens* (55 lots, 12.6 %) and *E. caballus* (54 lots, 12.3 %), followed by *Hydrochoerus hydrochaeris* (Linnaeus, 1766) (47 lots, 10.7 %), *T. terrestris* (46 lots, 10.5 %), *M. tridactyla* (26 lots, 5.9 %), *Panthera onca* (Linnaeus, 1758) (23 lots, 5.3 %), and *C. lupus familiaris* (21 lots, 4.8 %) (Additional file 1: Table S1).

### Ticks collected during field expeditions

A total of 1,746 ticks from 77 localities were collected during the three field expeditions. These were morphologically identified as 249 females of *A. cajennense* (*s.s.*), 443 females of *A. sculptum* and 1,054 males of *A. cajennense* (*s.l.*) Detailed information on the ticks collected at each locality are listed in Table 1. Some of the above male specimens were identified to *A. cajennense* (*s.s.*) or *A. sculptum* by molecular methods, as stated in Table 2. At least 23 localities contained the presence of both *A. cajennense* (*s.s.*) and *A. sculptum* in sympatry, inferred by the presence of females of both species co-infesting the same individual hosts.

**Table 1:** Data for the ticks *Amblyomma cajennense* (*sensu stricto*) [*A.c*.(*s.s.*)], *Amblyomma sculptum* (*A.s*.) and *Amblyomma cajennense* (*sensu lato*) [*A.c*.(*s.l.*)] collected during three field expeditions in Brazil^a^

Exp.	Number of ticks			Host	Municipality	State	Geographical coordinates (S, W)
	*A.c*.(*s.s*.)	*A.s.*	*A.c.*(*s.l*.)				
	♀♀	♀♀	♂♂				
1st			1	*Equus caballus*	Cach. do Piriá	Pará	1°50.888′, 45°23.452′
1st	21		59	*E. caballus*	S. G. Araguaia	Pará	5°59.592′, 48°37.947′
1st	1	1	1	*E. caballus*	S. G. Araguaia	Pará	6°06.594′, 48°35.672′
1st	2	8	7	*E. caballus*	Alvorada	Tocantins	12°23.111′, 49°07.061′
1st	3	3	6	*E. caballus*	Araguaína	Tocantins	7°19.570′, 48°18.255′
1st	7		10	*E. caballus*	Araguanã	Tocantins	6°35.582′, 48°38.493′
1st	3			*E. caballus*	Araguanã	Tocantins	6°51.336′, 48°30.986′
1st		12	25	*E. caballus*	Araguatins	Tocantins	5°45.278′, 48°03.427′
1st		1	4	*E. caballus*	Barrolândia	Tocantins	9°44.843′, 48°42.105′
1st	1	10	12	*E. caballus*	Barrolândia	Tocantins	9°51.657′, 48°43.446′
1st	1	1	2	Free-living	Bras. Tocantins	Tocantins	8°29.058′, 48°29.054′
1st	1	2	3	*E. caballus*	Brej. Nazaré	Tocantins	11°02.660′, 48°45.039′
1st		1	19	*E. caballus*	Brej. Nazaré	Tocantins	10°59.359′, 48°33.451′
1st	5	18	44	*E. caballus*	Cariri Tocantins	Tocantins	11°53.841′, 49°10.020′
1st	1	6	7	*E. caballus*	Crix. Tocantins	Tocantins	11°12.863′, 48°55.806′
1st	4	74	141	*E. caballus*	Figueirópolis	Tocantins	12°13.940′, 49°09.919′
1st		6	32	*Sus scrofa*	Fort. Tabocão	Tocantins	9°07.003′, 48°32.006′
1st	2	18	17	*E. caballus*	Guaraí	Tocantins	8°58.520′, 48°29.955′
1st		9	11	*E. caballus*	Gurupi	Tocantins	11°33.700′, 49°01.503′
1st	5	9	19	*E. caballus*	Gurupi	Tocantins	11°49.618′, 49°07.801′
1st		6	11	*E. caballus*	Luzinópolis	Tocantins	6°10.583′, 47°51.664′
1st	8	5	27	*Myrmecophaga tridactyla*	Miranorte	Tocantins	9°24.910′, 48°34.253′
1st		14	16	*E. caballus*	Nazaré	Tocantins	6°23.002′, 47°42.193′
1st	1		3	*E. caballus*	Nova Olinda	Tocantins	7°47.974′, 48°27.873′
1st	1	4	1	*E. caballus*	N. Rosalândia	Tocantins	10°34.032′, 48°55.116′
1st		3	1	*E. caballus*	Par. Tocantins	Tocantins	10°07.183′, 48°52.998′
1st		2	10	*E. caballus*	Porto Nacional	Tocantins	10°29.502′, 48°20.153′
1st	7	4	45	*E. caballus*	S. R. Tocantins	Tocantins	10°54.404′, 48°54.798′
1st	1	2	1	*E. caballus*	S. B. Tocantins	Tocantins	5°58.436′, 47°52.737′
1st	1	1	24	*E. caballus*	Talismã	Tocantins	12°47.346′, 49°05.470′
1st	2	35	27	*E. caballus*	Tocantinópolis	Tocantins	6°15.035′, 47°27.741′
1st	1	3	6	*E. caballus*	Tocantinópolis	Tocantins	6°20.687′, 47°33.018′
1st	1			*E. caballus*	Xambioá	Tocantins	6°24.783′, 48°32.321′
1st			10	*E. caballus*	Bequimão	Maranhão	2°27.784′, 44°47.416′
1st	7		18	*S. scrofa*	B. Jesus Selvas	Maranhão	4°31.142′, 46°46.691′
1st			2	*E. caballus*	Imperatriz	Maranhão	5°33.209′, 47°27.766′
1st			3	*E. caballus*	Pinheiro	Maranhão	2°33.518′, 59°47.764′
1st	2		2	*E. caballus*	Porto Franco	Maranhão	6°20.177′, 47°24.480′
1st	1		7	*E. caballus*	Santa Helena	Maranhão	2°15.681′, 45°16.235′
1st			4	*E. caballus*	Santa Inês	Maranhão	3°41.666′, 45°24.301′
1st	9		7	*E. caballus*	Santa Inês	Maranhão	3°52.575′, 45°32.757′
1st	7		5	*E. caballus*	S. Luzia do Tide	Maranhão	4°04.668′, 45°57.052′
1st		4	2	*E. caballus*	Estrela Norte	Goiás	13°49.376′, 49°01.775′
1st	1	2	2	*E. caballus*	Porangatu	Goiás	12°52.722′, 49°06.359′
1st		5	5	*E. caballus*	Porangatu	Goiás	12°53.209′, 49°06.390′
1st		6	21	*E. caballus*	Porangatu	Goiás	13°24.868′, 49°08.118′
1st		1	4	*E. caballus*	Porangatu	Goiás	13°15.364′, 49°08.122′
1st		7	18	*E. caballus*	S. Ter. Goiás	Goiás	13°39.022′, 49°02.094′
2nd	5	12	7	*E. caballus*	Pimenta Bueno	Rondônia	11°52.328′, 60°59.868′
2nd	2		1	*E. caballus*	Presid. Médici	Rondônia	11°09.754′, 61°54.095′
2nd	2			Free-living	Vilhena	Rondônia	12°43.412′, 60°15.417′
2nd	1		4	Free-living	Vilhena	Rondônia	12°29.545′, 60°28.309′
2nd	5		6	*E. caballus*	Comodoro	Mato Grosso	13°56.276′, 59°45.634′
2nd	32		7	Free-living	Comodoro	Mato Grosso	13°56.276′, 59°45.634′
2nd	2			Free-living	Comodoro	Mato Grosso	13°19.058′, 59°52.654′
2nd			1	Free-living	Comodoro	Mato Grosso	13°05.216′, 59°53.509′
2nd	4		3	Free-living	Comodoro	Mato Grosso	13°00.277′, 59°57.472′
2nd	1	49	26	*E. caballus*	Conq. d’Oeste	Mato Grosso	14°46.905′, 59°20.774′
2nd	4		6	*E. caballus*	Nova Lacerda	Mato Grosso	14°10.232′, 59°42.056′
2nd	1		1	Free-living	Nova Lacerda	Mato Grosso	14°07.560′, 59°42.607′
2nd			1	*E. caballus*	Pont. Lacerda	Mato Grosso	15°09.292′, 59°28.354′
2nd		4	3	*E. caballus*	Pont. Lacerda	Mato Grosso	15°20.685′, 59°23.777′
2nd		3		*E. caballus*	Pont. Lacerda	Mato Grosso	14°53.271′, 59°16.735′
2nd		3	3	*E. caballus*	V. B. S. Trindade	Mato Grosso	15°07.393′, 59°34.550′
2nd		1	1	*E. caballus*	V. B. S. Trindade	Mato Grosso	15°00.374′, 59°57.109′
2nd		5	11	*E. caballus*	V. B. S. Trindade	Mato Grosso	14°58.490′, 59°59.009′
2nd	1	31	31	Free-living	V. B. S. Trindade	Mato Grosso	14°55.576′, 60°01.216′
2nd		11	11	Free-living	V. B. S. Trindade	Mato Grosso	15°05.634′, 59°51.829′
3rd		10	34	*E. caballus*	Diamantino	Mato Grosso	14°33.153′, 56°13.418′
3rd		2		*E. caballus*	Jangada	Mato Grosso	15°48.708′, 56°39.218′
3rd	3	1	7	*E. caballus*	Lucas Rio Verde	Mato Grosso	13°45.656′, 55°52.487′
3rd		8	9	*E. caballus*	Nobres	Mato Grosso	14°43.526′, 56°19.525′
3rd		8	3	*E. caballus*	Rosário Oeste	Mato Grosso	14°48.500′, 56°25.580′
3rd		12	11	*E. caballus*	Rosário Oeste	Mato Grosso	14°48.408′, 56°25.278′
3rd			21	*E. caballus*	Sinop	Mato Grosso	11°52.466′, 55°36.316′
3rd	11		7	*E. caballus*	Sinop	Mato Grosso	11°55.516′, 55°42.000′
3rd	68		137	*Tapirus terrestris*	Sinop	Mato Grosso	13°45.660′, 55°52.487′
Total	249	443	1,054				

Exp.: Field expedition

^a^ 1st expedition: considered to represent sampling in the eastern transition area of *A. cajennense* (*s.s.*) and *A. sculptum.* 2nd expedition: considered to represent sampling in the western transition area of the two tick species in Brazil. 3rd expedition: considered to represent the central transition area of the two species in Brazil

**Table 2:** Data for the ticks *Amblyomma cajennense* (*sensu stricto*) (*s.s.*) and *Amblyomma sculptum* from Brazil used for molecular and phylogenetic analyses in the present study

Tick species	Gender	Host	Date	Locality			No. of specimens	Haplotype code
				Municipality	State	Geographical Coordinates (S, W)		
*A. cajennense* (*s.s.*)	♂	*Equus caballus*	Jan/2012	Cachoeira do Piriá	Pará	1°50′, 45°23′	1	A
*A. cajennense* (*s.s.*)	♂	*E. caballus*	Oct/2011	S. Dom. Capim	Pará	1°40′, 47°46′	1	A
*A. cajennense* (*s.s.*)	♀	*E. caballus*	Jan/2012	S. Ger. Araguaia	Pará	6°6′, 48°35′	2	A
*A. cajennense* (*s.s.*)	♂	*Bubalus bubalis*	Sep/2011	S. Franc. Guaporé	Rondônia	12°3′, 63°34′	2	A
*A. cajennense* (*s.s.*)	♀	Free-living	Mar/2002	Vilhena	Rondônia	12°44′, 60°8′	3	A
*A. cajennense* (*s.s.*)	♀^a^	*Canis familiaris*	Aug/2011	Cristalândia	Tocantins	10°36′, 49°11′	2	A
*A. cajennense* (*s.s.*)	♂	*E. caballus*	Jan/2012	Bequimão	Maranhão	2°27′, 44°47′	2	A
*A. cajennense* (*s.s.*)	♂	*E. caballus*	Jan/2012	Chapadinha	Maranhão	3°44′, 43°21′	2	A
*A. cajennense* (*s.s.*)	♂	*E. caballus*	Jan/2012	Pinheiro	Maranhão	2°33′, 59°47′	2	A
*A. cajennense* (*s.s.*)	♂	*E. caballus*	Jan/2012	Santa Inês	Maranhão	3°41′, 45°24′	2	A
*A. cajennense* (*s.s.*)	♂	Free-living	Jan/2012	Comodoro	Mato Grosso	13°5′, 59°53′	1	A
*A. cajennense* (*s.s.*)	♂	*E. caballus*	Apr/2011	Confresa	Mato Grosso	10°38′, 51°34′	2	A
*A. cajennense* (*s.s.*)	♂	*E. caballus*	Jan/2012	Pontes e Lacerda	Mato Grosso	15°9′, 59°28′	1	A
*A. cajennense* (*s.s.*)	♂	*E. caballus*	Feb/2013	Sinop	Mato Grosso	11°52′, 55°36′	1	A
*A. sculptum*	♀	*E. caballus*	Jan/2012	Pimenta Bueno	Rondônia	11°52′, 60°59′	2	B, C
*A. sculptum*	♂	Free-living	Jun/2011	Tocantinópolis	Tocantins	6°19′, 47°24′	2	D
*A. sculptum*	♂	*E. caballus*	Aug/2012	Campo Formoso	Bahia	10°30′, 40°19′	1	E
*A. sculptum*	♂	*E. caballus*	Jul/2011	Balsas	Maranhão	7°31′, 46°2′	2	E, F
*A. sculptum*	♂	*E. caballus*	Jan/2012	Imperatriz	Maranhão	5°33′, 47°27′	1	G
*A. sculptum*	♂	*Equus asinus*	Jan/2011	José de Freitas	Piauí	4°45′, 42°34′	2	E
*A. sculptum*	♂	*E. caballus*	Jun/2011	Cumari	Goiás	18°15′, 48°9′	1	E
*A. sculptum*	♂	*E. caballus*	Jan/2012	Porangatu	Goiás	12°52′, 49°6′	1	E
*A. sculptum*	♂	*E. caballus*	Jul/2011	Poconé	Mato Grosso	16°15′, 56°37′	1	H
*A. sculptum*	♂	*Sus scrofa*	Aug/2010	Corumbá	M. Grosso do Sul	19°0′, 57°39′	2	E
*A. sculptum*	♂	*E. caballus*	Jul/2011	Pinheiros	Espírito Santo	18°24′, 40°13′	1	E
*A. sculptum*	♂	*E. caballus*	Jan/2011	Itabira	Minas Gerais	19°37′, 43°13′	2	E
*A. sculptum*	♂	*E. caballus*	May/2011	Seropédica	Rio de Janeiro	22°44′, 43°42′	2	E
*A. sculptum*	♂	Free-living	Oct/2011	Americana	São Paulo	22°44′, 47°19′	2	E
*A. sculptum*	♂	*E. caballus*	Dec/2008	Pirassununga	São Paulo	21°59′, 47°25′	3	E
*A. sculptum*	♂	*E. caballus*	May/2012	Alvorada do Sul	Paraná	22°46′, 51°13′	1	E
Total							50	

^a^ This tick was collected as an engorged nymph, and molted to the adult stage in the laboratory

### Molecular and phylogenetic analyses

**Fig. 2:**
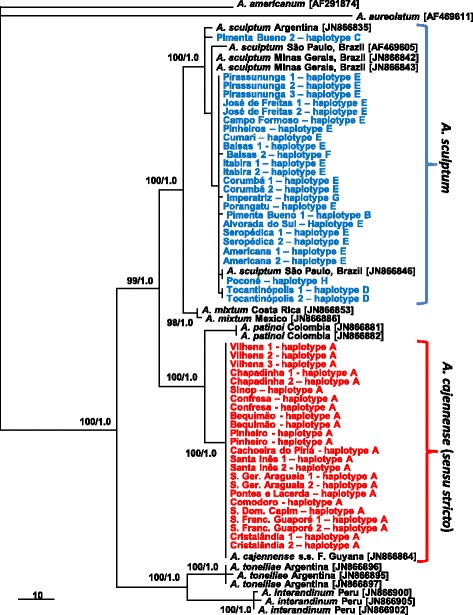
Bayesian inference phylogenetic tree with Maximum parsimony (MP) support values of the ITS2 rDNA sequences generated in the present study for the ticks *Amblyomma cajennense* (*sensu lato*), including 24 genotypes of *A. cajennense* (*sensu stricto*), indicated in red, and 26 genotypes of *A. sculptum*, indicated in blue. Each genotype is labelled as: Municipality name, number of the tick specimen, and haplotype code, as shown in Table 2. ITS2 sequences for *Amblyomma americanum* and *A. aureolatum* were used as the outgroup. Numbers at nodes are support values derived from bootstrap (1,000 replicates for MP/posterior probabilities for Bayesian inference analysis). Numbers in brackets are GenBank accession numbers

DNA sequences of the ITS2 gene were generated for 24 *A. cajennense* (*s.s.*) from 14 different localities, and 26 *A. sculptum* from 16 localities (Table 2). Phylogenetic analyses of these ITS2 sequences with corresponding sequences from GenBank formed six main clades, each corresponding to a species of *A. cajennense* (*s.l*.): *A. cajennense* (*s.s.*), *A. sculptum*, *A. mixtum*, *A. tonelliae*, *A. internadinum* and *A. patinoi* (Fig. 2). There was no polymorphism among the *A. cajennense* (*s.s.*) sequences, since all 24 tick specimens shared the same haplotype, which was 100 % identical to the GenBank ITS2 sequence of *A. cajennense* (*s.s.*) from French Guyana (JN866864). Conversely, the 26 *A. sculptum* presented polymorphism, as they yielded seven distinct ITS2 haplotypes. This polymorphism was extended to the *A. sculptum* sequences from GenBank, as they also represented distinct haplotypes in the phylogenetic tree (Fig. 2). The haplotypes of *A. cajennense* (*s.s.*) (haplotype A) and *A. sculptum* (haplotypes B-H) generated in the present study have been submitted to GenBank under the accession numbers KU169881-KU169888.

### Distribution maps

**Fig. 3:**
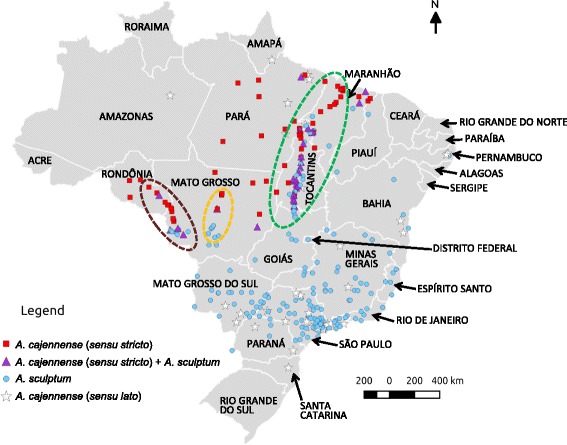
Geopolitical map of Brazil, showing the localities of *Amblyomma cajennense* (*sensu stricto*), *A. sculptum* and *A. cajennense* (*sensu lato*) identified in the present study. Dashed line ellipses include the localities that were sampled during three field expeditions for tick collections, the first expedition in green, the second in brown and the third in orange

**Fig. 4:**
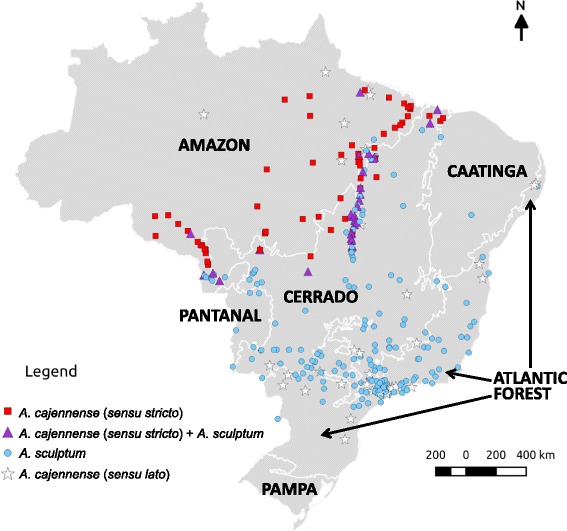
Map of Brazil showing the localities of the ticks *Amblyomma cajennense* (*sensu stricto*), *A. sculptum* and *A. cajennense* (*sensu lato*) identified in the present study, according to the six major biomes (Amazon, Atlantic forest, Caatinga, Cerrado, Pantanal, Pampa) that compose the Brazilian land

**Fig. 5:**
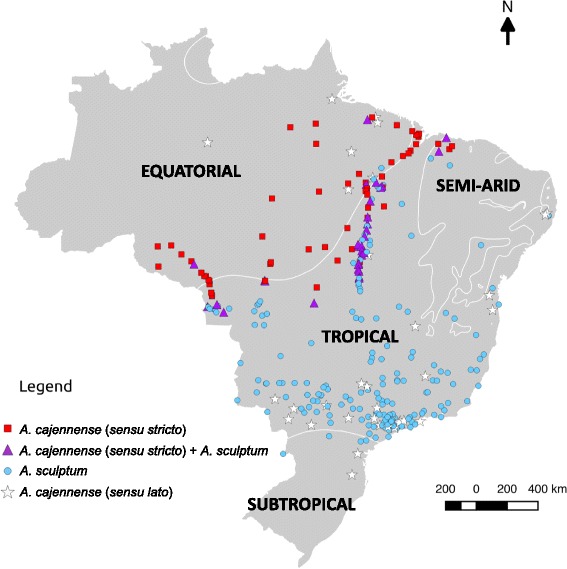
Map of Brazil showing the localities of the ticks *Amblyomma cajennense* (*sensu stricto*), *A. sculptum* and *A. cajennense* (*sensu lato*) identified in the present study, according to the four major climates (Equatorial, Tropical, Semi-arid, and Subtropical) that occur in the Brazilian land

**Fig. 6:**
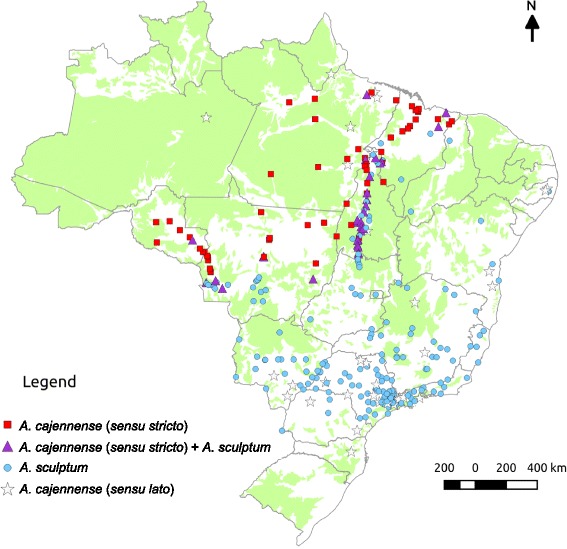
Map of Brazil showing the localities of the ticks *Amblyomma cajennense* (*sensu stricto*), *A. sculptum,* and *A. cajennense* (*sensu lato*) identified in the present study, according to natural vegetation cover, represented in green

A total of 58 localities with the occurrence of only *A. cajennense* (*s.s.*), 184 for only *A. sculptum,* 31 for both *A. cajennense* (*s.s.*) and *A. sculptum*, and 36 for *A. cajennense* (*s.l.*) (only male specimens) were used to construct distribution maps according to geopolitical regions (Fig. 3), biomes (Fig. 4), climate (Fig. 5) and natural vegetation cover (Fig. 6). The three field expeditions encompassed transition areas of the two species in Brazil, since almost all localities with sympatric occurrence of *A. cajennense* (*s.s.*) and *A. sculptum* were derived from these expeditions (Fig. 3). Therefore, these transition areas correspond primarily to the states of Maranhão and Tocantins in eastern Brazil, Mato Grosso in central Brazil, and Mato Grosso and Rondônia in western Brazil. These transition areas correspond to the geographic boundaries of the Amazon and Cerrado biomes (Fig. 4). In addition to these transition areas, *A. cajennense* (*s.s.*) was restricted to areas within the Amazon biome, whereas *A. sculptum* was found in the Cerrado, Pantanal, and Atlantic forest biomes. Interestingly, the single record of *A. sculptum* in the Caatinga biome (Fig. 4) was from an area of tropical climate, instead of the dominant semiarid climate of this biome (Fig. 5). The distribution of *A. cajennense* (*s.s.*) was almost restricted to areas with equatorial climate (which generally coincides with Amazon biome), whereas *A. sculptum* was almost restricted to areas with tropical climate (which generally coincides with Cerrado and Atlantic forest biomes) (Fig. 5). There were very few records of *A. sculptum* or *A. cajennense* (*s.l.*) from areas with subtropical climate in southern Brazil. In addition, the Pampa biome (southernmost Brazil) was the only biome where *A. cajennense* (*s.l.*) was absent in Brazil (Fig. 4). In the Amazon biome, the occurrence of *A. cajennense* (*s.s.*) was generally restricted to areas where the natural vegetation cover was absent. The same applies to *A. sculptum* in the Atlantic forest biome. On the other hand, in the Cerrado and Pantanal biomes, *A. sculptum* was found in areas with or without natural vegetation cover (Fig. 6).

### Description of the nymph of *A. cajennense* (*s.s.*)

**Fig. 7:**
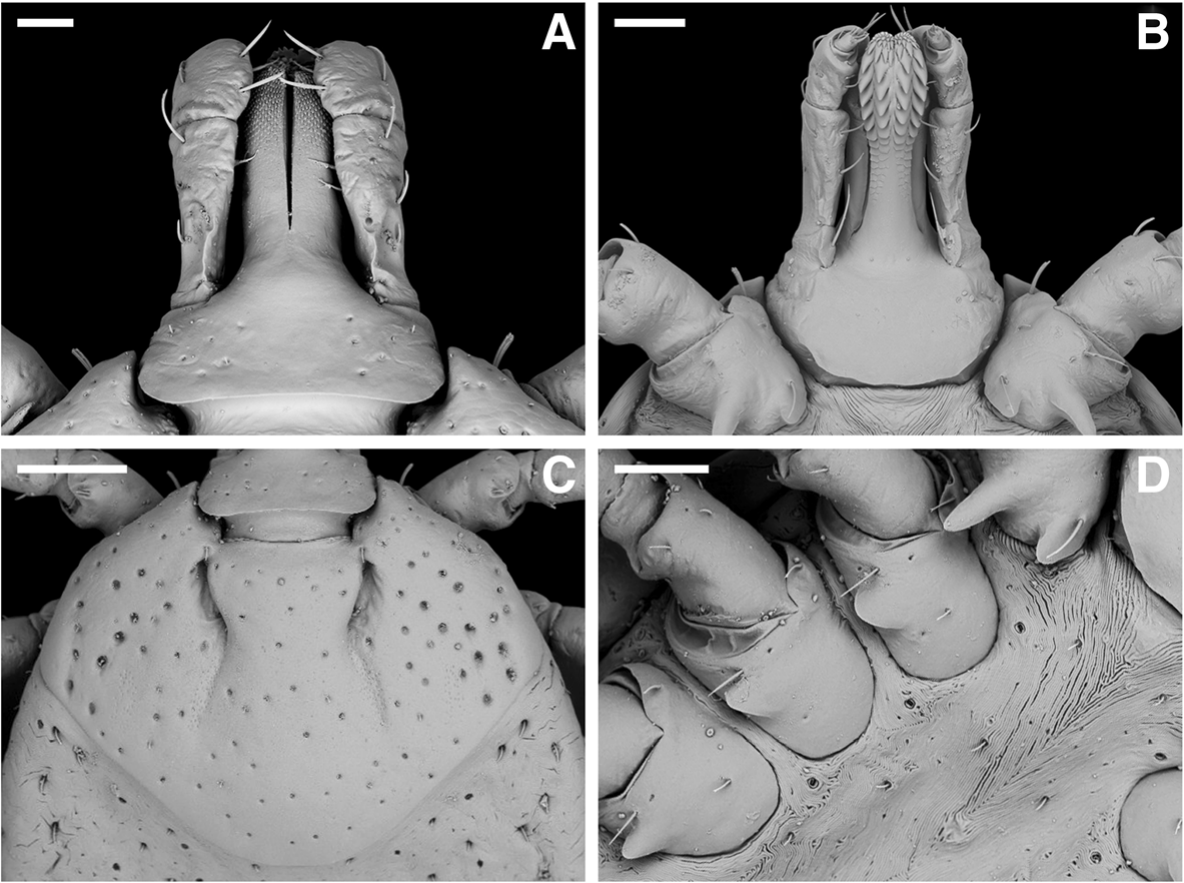
*Amblyomma cajennense* (*sensu stricto*) nymph. **a** Dorsal gnathosoma (capitulum); **b** Ventral gnathosoma; **c** Scutum; **d** Coxae I–IV. *Scale-bars*: a, 60 μm; b, d, 100 μm; c, 200 μm

[Based on 10 specimens; (Fig. 7).] *Idiosoma.* Length from apices of scapula to posterior body margin: 1,280–1,579 (1,444); maximum breadth 1,040–1,257 (1,156); outline oval, with 11 festoons without tubercles. Scutum 681–784 (730) long, 835–944 (887) wide, breadth/length ratio 1.146–1.265 (1.214), inornate; large and deep punctations evenly distributed. Eyes not orbited at lateral scutal angles at level of scutal midlength. Cervical grooves deep in scutal anterior third, followed by rugose shallow depression in scutal median third. Spiracular plate triangular with rounded angles, with an evident dorsal prolongation, 256–317 (276) long, 192–257 (218) wide.

*Gnathosoma.* Length from palpal apices to dorsal posterior margin 373–473 (414), breadth 317–353 (331). Basis capituli rectangular, posterior margin slightly concave, without cornua; posterior margin convex ventrally, without auriculae. Palpi length 280–345 (307), article I with vestigial ventral prolongation, article II 165–208 (182) long, article III 86–116 (98) long. Hypostome rounded apically, 291–364 (316) long; length of toothed portion 164–204 (178); dentition 2/2 with 7–8 teeth per row.

*Legs.* Coxa I with 2 pointed spurs separated by space equal or greater than breadth of external spur base; external spur about 2× longer than internal; coxae II–IV with small triangular spur. Trochanters without spur; tarsus I 431–509 (450) long, 99–115 (109) wide; tarsus IV 320–372 (337) long, 72–99 (86) wide.

*Remark*: The nymph of *A. cajennense* (*s.s.*) is morphologically very similar to the nymph of *A. sculptum*, recently described [7]. Comparing the two descriptions, the only concrete difference is the scutal width/length ratio [< 1.3 in *A. cajennense* (*s.s*.) *vs* > 1.3 in *A. sculptum*]. *Amblyomma cajennense* (*s.s.*) can be separated from *A. tonelliae* by using the same criteria recently proposed to separate *A. tonelliae* from *A. sculptum*, based on scutum surface and the length of the antero-lateral seta on coxa I [8].

### Discussion

In the present study, examination of all lots of *A. cajennense* (*s.l.*) from two large tick collections of Brazil, plus collection of new material during three field expeditions, confirmed that *A. cajennense* (*s.l.*) is currently represented in Brazil by only two species, *A. cajennense* (*s.s.*) and *A. sculptum.* This result was corroborated by molecular analyses inferred from the ITS2 ribosomal gene. We chose this molecular marker because a recent study revealed that the ITS2 gene fragment is suitable for evolutionary studies on *A. cajennense* [5]. If any other species of *A. cajennense* (*s.l.*) occurs in Brazil, it might have a much more restricted distribution, since our analyses encompassed areas throughout the Brazilian land.

As previously reported [2, 5], our study confirms that *A. cajennense* (*s.s.*) occurs generally in the Amazon biome; however, almost all records were from the edge of this biome, among the so called “Seasonally Dry Tropical Forest” (SDTF) part of the Amazon biome; i.e., this tick is not found in thick of the rain forest [5], which generally means the inner part of the forest. While the absence of records from areas deeper inside the Amazon biome could be related to poor sampling in these less accessible areas, it is noteworthy that an extensive study in the state of Rondônia (Brazilian western Amazon) made multiple samplings throughout the state, and concluded that *A. cajennense* was generally absent from areas with dense Amazon forest [21]. The above statements are corroborated by our results shown in Fig. 6, where all records of *A. cajennense* (*s.s.*) are within areas with degraded forest cover, i.e. this species was not found at any area where natural vegetation cover remains in the Amazon biome. This geographic distribution could be related to particular abiotic conditions required by *A. cajennense* (*s.s.*), suggesting that this tick might not be adapted to extremely humid rainforests. On the other hand, the absence of *A. cajennense* (*s.s.*) deeper in the Cerrado biome (Brazilian savannah) could be related to unsuitable abiotic conditions at the opposite extreme, namely, the much drier seasonally drought period, typical for the tropical climate that prevails in the Cerrado (Figs. 3, 4).

The distribution of *A. sculptum* in Brazil generally encompassed entire Cerrado and Pantanal biomes and great part of the Atlantic forest biome (Fig. 3). This distribution coincides with the tropical climate. Interestingly, the absence of *A. sculptum* in southern Brazil (Fig. 2) coincides with the subtropical climate (Fig. 4), suggesting that the cooler temperatures during autumn/winter in this region might be the limiting factor for the establishment of *A. sculptum*. This assumption is corroborated by previous modelling studies [1, 22]. While *A. sculptum* seems to be very adapted to the Cerrado biome, the same cannot be applied to the Caatinga biome, where the semiarid climate (Figs. 2, 3) is possibly the main limiting factor. Interestingly, our single record of *A. sculptum* in the Caatinga biome was from the state of Bahia (Figs. 2, 3), in an area that represents a narrow “invagination” of the tropical climate into the Caatinga (Fig. 4).

It is noteworthy that nearly all records of *A. sculptum* in the Atlantic forest biome were from areas where the natural vegetation cover has been degraded (Fig. 5), i.e. this tick was not found at any area where natural vegetation cover remains in the Atlantic forest biome. This result is corroborated by an extensive field study in a large Atlantic forest Reserve in Southern São Paulo, where *A. sculptum* (reported as *A. cajennense*) was never found inside the dense forest, where suitable hosts (e.g. tapirs) were abundant; on the other hand, *A. sculptum* was abundant in an open, degraded area within the Reserve [23]. The authors suggested that the distribution of *A. sculptum* has increased as a result of destruction of the Atlantic rainforest over the last few centuries. In fact, the Atlantic forest biome is indeed the most degraded Brazilian biome, which retains < 10 % of its original natural vegetation cover [24]. This condition can be clearly observed in Figs. 2 and 5. It seems likely that abiotic factors of these degraded areas of the Atlantic forest “simulate” the conditions of the Cerrado biome, where *A. sculptum* prevails on either natural or degraded areas, as shown in Fig. 5. This statement is corroborated by a previous study with the use of satellite imagery and the distribution of *A. cajennense* (*s.l.*) [1], which showed that both the Cerrado and the degraded Atlantic forest areas of Brazil presented similar normalised derived vegetation index (NVDI). This index is an indirect quantification of abiotic factors.

In the present study, we were able to find three transition areas of the distribution of *A. cajennense* (*s.s.*) and *A. sculptum* in Brazil, where these tick species were sympatric (Fig. 2). These results are in agreement with a recent environmental modelling study suggesting that *A. cajennense* (*s.s.*) and *A. sculptum* may overlap in parts of their range due to some similarities in abiotic variables [22]. Because these transition areas corresponded to the boundaries between the Amazon and Cerrado biome, or between the equatorial and tropical climate, we can infer that these transition areas represent extreme abiotic conditions for the distribution of the two species.

Continuing degradation of the Amazon forest, with replacement of the original forest cover by Cerrado-like vegetation cover may favour the expansion of *A. sculptum* into these areas. A potential example of this condition is the state of Rondônia, where Labruna et al. [21] did not find any *A. sculptum* during a large, extensive tick survey throughout the state during 2000–2005. Nearly one decade later, in 2012 (during our second field expedition), we were able to find an established population of *A. sculptum* infesting horses in Pimenta Bueno municipality (corresponds to the single record of *A. sculptum* in Rondônia in Fig. 2). Because Rondônia is one of the Brazilian states with the highest deforestation and forest degradation indices during the last two decades [25], this condition might have facilitated the expansion of *A. sculptum* into the state. This scenario has extreme medical relevance, since in Brazil *A. sculptum* is the most important vector of the bacterium *Rickettsia rickettsii*, the etiological agent of the Brazilian spotted fever, the most lethal spotted fever of the world [26]. On the other hand, *A. cajennense* (*s.s.*) has only been found infected by *Rickettsia amblyommii*, a much less or non-pathogenic rickettsial agent [27]. Hence, expansion of *A. sculptum* into northern Brazil could involve expansion of other tick-borne diseases, especially because *A. sculptum* is indeed the most common human-biting tick in Brazil [28]. Our data corroborate this assumption, since *H. sapiens* was the most frequent host species for *A. sculptum* among the two tick collections examined in the present study.

### Conclusions

Results of the present study confirmed that *A. cajennense* (*s.l.*) is currently represented in Brazil by only two species, *A. cajennense* (*s.s.*) and *A. sculptum.* While the two species have distinct distribution areas in the country, they are found in sympatry in some transition areas. The current distribution of *A. cajennense* (*s.l.*) has important implications to public health, since in Brazil *A. sculptum* is the most important vector of the bacterium *R. rickettsii*, the etiological agent of the most lethal spotted fever of the world, the Brazilian spotted fever.

## Additional file

Additional file 1: Table S1. Records of *Amblyomma cajennense sensu stricto* (*s.s.*), *Amblyomma sculptum,* and *Amblyomma cajennense sensu lato* (*s.l.*) that were examined in the tick collections “Acari Colletion of the Instituto Butantan” (IBSP), São Paulo, Brazil, and “Coleção Nacional de Carrapatos” (CNC) of the University of São Paulo, São Paulo, Brazil. (PDF 1091 kb)
