# Supplementary material for: Geographical distribution of Amblyomma cajennense (sensu lato) ticks (Parasitiformes: Ixodidae) in Brazil, with description of the nymph of A. cajennense (sensu stricto)
Source: Parasit Vectors. 2016 Mar 31;9:186. doi: 10.1186/s13071-016-1460-2 (PMC4818509; doi:10.1186/s13071-016-1460-2)
Supplement: Additional file 1: Table S1. — Records of Amblyomma cajennense sensu stricto (s.s.), Amblyomma sculptum, and Amblyomma cajennense sensu lato (s.l.) that were examined in the tick collections “Acari Colletion of the Instituto Butantan” (IBSP), São Paulo, Brazil, and “Coleção Nacional de Carrapatos” (CNC) of the University of São Paulo, São Paulo, Brazil. (PDF 1091 kb) [file 13071_2016_1460_MOESM1_ESM.pdf]

Supplemental Table 1: Records of *Amblyomma cajennense sensu stricto* (*s.s.*), *Amblyomma sculptum*, and *Amblyomma cajennense sensu lato* (*s.l.*) that were examined in the tick collections “Acari Colletion of the Instituto Butantan” (IBSP), São Paulo, Brazil, and “Coleção Nacional de Carrapatos” (CNC) of the University of São Paulo, São Paulo, Brazil.

Tick species	No. Males **	No. Females	host	municipality	state	latitude longitude	date	Acession number
**IBSP**								
A. cajennense s. s		1	*T. terrestris*	Altamira	Pará	3°12’S 52°12’W	March 1939	IBSP 1661
A. cajennense s. s	4	3	*T. terrestris*	Altamira	Pará	3°12’S 52°12’W	March 1939	IBSP 1665
A. cajennense s. s.	5	3	*T. terrestris*	Nova Xavantina	Mato Grosso	14°40’S 52°21’W	February 1952	IBSP 3867
A. cajennense s. s.	1	2	*E. caballus*	Monte Alegre	Pará	2°0’S 54°4’W	August 1957	IBSP 5196
A. cajennense s. s.	29	16	*H. sapiens*	Monte Alegre	Pará	2°0’S 54°4’W	August 1957	IBSP 5179
A. cajennense s. s.	4	8	*H. sapiens*	Monte Alegre	Pará	2°0’S 54°4’W	August 1957	IBSP 5193
A. cajennense s. s.	1	3	*T. terrestris*	Monte Alegre	Pará	2°0’S 54°4’W	August 1957	IBSP 5186
A. cajennense s. s.	2	3	*T. terrestris*	Novo Progresso	Pará	7°8’S 55°22’W	March 1958	IBSP 5641
A. cajennense s. s.		1	unknown	Parauapebas	Pará	6°4’S 49°54’W	September 2012	IBSP 11590
A. cajennense s. s.		1	unknown	Parauapebas	Pará	6°4’S 49°54’W	September 2012	IBSP 11597
A. cajennense s. s.		2	*C. denticulata*	São Félix do Xingu	Pará	6°38’S 51°59’W	April 1999	IBSP 7735
A. cajennense s. s.	1	1	*C. denticulata*	São Félix do Xingu	Pará	6°38’S 51°59’W	April 1999	IBSP 7749
A. cajennense s. s.	2	1	free living	São Félix do Xingu	Pará	6°38’S 51°59’W	April 1999	IBSP 7751
A. cajennense s. s.		1	free living	Pimenta Bueno	Rondônia	11°40’S 61°11’W	July 2000	IBSP 7794
A. cajennense s. s.		3	free living	Canarana	Mato Grosso	13°33’S 52°16’W	December 2000	IBSP 7627
A. cajennense s. s.	8	3	*T. terrestris*	São José do Xingu	Mato Grosso	10°48’S 52°44’W	March 1958	IBSP 5644
A. cajennense s. s. *		1	*C. familiaris*	São José do Xingu	Mato Grosso	10°48’S 52°44’W	October 1969	IBSP 5951
A. cajennense s. s. *	8	14	*C. familiaris*	Nova Xavantina	Mato Grosso	14°40’S 52°21’W	September 1969	IBSP 5950
A. sculptum	1	1	*E. caballus*	Belém	Pará	1°27’S 48°30’W	September 1998	IBSP 6860
A. sculptum	1	1	*C. thous*	Cuiabá	Mato Grosso	15°35’S 56°5’W	September 1952	IBSP 4598
A. sculptum		1	*I.iguana*	Cuiabá	Mato Grosso	15°35’S 56°5’W	April 1997	IBSP 7702
A. sculptum	3	5	*M. tridactyla*	Cuiabá	Mato Grosso	15°35’S 56°5’W	September 2006	IBSP 7703
A. sculptum	8	11	*T. terrestris*	Cuiabá	Mato Grosso	15°35’S 56°5’W	November 1996	IBSP 7710
A. sculptum	2	1	unknown	Batayporã	Mato Grosso do Sul	22°17’S 53°16’W	February 2001	IBSP 7308
A. sculptum	14	18	free living	Bonito	Mato Grosso do Sul	21°7’S 56°28’W	October 2002	IBSP 8929
A. sculptum	10	10	free living	Bonito	Mato Grosso do Sul	21°7’S 56°28’W	October 2002	IBSP 8930
A. sculptum	1	10	free living	Bonito	Mato Grosso do Sul	21°7’S 56°28’W	October 2002	IBSP 8931
A. sculptum	14	12	free living	Bonito	Mato Grosso do Sul	21°7’S 56°28’W	October 2002	IBSP 8932
A. sculptum		1	free living	Corumbá	Mato Grosso do Sul	19°0’S 57°39’W	January 2000	IBSP 7634
A. sculptum		1	unknown	Coxim	Mato Grosso do Sul	18°30’S 54°45’W	June 1952	IBSP 4532
A. sculptum	2	1	*T. tetradactyla*	Formoso	Minas Gerais	14°56’S 46°13’W	October 1952	IBSP 4582
A. sculptum		1	free living	Itabirito	Minas Gerais	20°15’S 43°48’W	April 2007	IBSP 9873

A. sculptum		1	free living	Taiobeiras	Minas Gerais	15°48'S 42°13'W	April 2002	IBSP 9932
A. sculptum		1	*B. torquatus*	Campos dos Goytacazes	Rio de Janeiro	21°45'S 41°19'W	February 1991	IBSP 7309
A. sculptum		1	*H. sapiens*	Águas da Prata	São Paulo	21°56'S 46°43'W	July 2004	IBSP 9062
A. sculptum		1	free living	Águas da Prata	São Paulo	21°56'S 46°43'W	August 2000	IBSP 7065
A. sculptum		1	free living	Águas da Prata	São Paulo	21°56'S 46°43'W	March 2003	IBSP 8757
A. sculptum		1	unknown	Águas de São Pedro	São Paulo	22°35'S 47°52'W	March 2006	IBSP 9730
A. sculptum		1	*H. sapiens*	Caieiras	São Paulo	23°21'S 46°44'W	November 2005	IBSP 9589
A. sculptum		1	*H. sapiens*	Caraguatatuba	São Paulo	23°37'S 45°24'W	December 2005	IBSP 9595
A. sculptum	3	10	*H. sapiens*	Caraguatatuba	São Paulo	23°37'S 45°24'W	December 2006	IBSP 9596
A. sculptum	5	3	free living	Bonito	Mato Grosso do Sul	21°7'S 56°28'W	October 2002	IBSP 8934
A. sculptum	5	3	*T. terrestris*	Formoso do Araguaia	Tocantins	11°47'S 49°31'W	December 1953	IBSP 4946
A. sculptum	1	3	free living	Palmas	Tocantins	10°11'S 48°20'W	June 2001	IBSP 7349
A. sculptum		1	free living	Ilhéus	Bahia	14°47'S 39°2'W	January 1999	IBSP 7614
A. sculptum	5	7	*F. catus*	Timbiras	Maranhão	4°15'S 43°56'W	February 1952	IBSP 4541
A. sculptum		1	*H. sapiens*	Brasília	Distrito Federal	15°47'S 47°52'W	May 1998	IBSP 6895
A. sculptum		1	*H. sapiens*	Ceres	Goiás	15°18'S 49°35'W	October 1934	IBSP 773
A. sculptum	1	3	unknown	Jaraguá	Goiás	15°45'S 49°20'W	August 1934	IBSP 768
A. sculptum	4	9	*H. hydrochaeris*	Pirenópolis	Goiás	15°51'S 48°57'W	May 1934	IBSP 769
A. sculptum		1	*D. albiventris*	Campo Grande	Mato Grosso do Sul	20°26'S 54°38'W	July 1997	IBSP 6888
A. sculptum	1	4	*E. caballus*	Corumbá	Mato Grosso do Sul	19°0'S 57°39'W	January 1998	IBSP 7030
A. sculptum	9	18	*T. tetradactyla*	Miranda	Mato Grosso do Sul	20°14'S 56°22'W	September 1952	IBSP 4557
A. sculptum		1	*T. tetradactyla*	Ponta Porã	Mato Grosso do Sul	22°32'S 55°43' W	September 1952	IBSP 4549
A. sculptum	21	21	*T. terrestris*	Porto Murtinho	Mato Grosso do Sul	21°41'S 57°52'W	October 1952	IBSP 4962
A. sculptum	14	1	*M. tridactyla*	Ribas do Rio Pardo	Mato Grosso do Sul	20°26'S 53°45'W	October 1952	IBSP 4544
A. sculptum	9	2	*M. tridactyla*	Ribas do Rio Pardo	Mato Grosso do Sul	20°26'S 53°45'W	October 1952	IBSP 4547
A. sculptum	4	1	*M. tridactyla*	Ribas do Rio Pardo	Mato Grosso do Sul	20°26'S 53°45'W	September 1952	IBSP 4554
A. sculptum	3	1	*P. onca*	Mato Verde	Minas Gerais	15°23'S 42°51'W	April 1950	IBSP 4538
A. sculptum	1	1	unknown	Morada Nova de Minas	Minas Gerais	18°36'S 45°21'W	January 2009	IBSP 10848
A. sculptum		1	unknown	Ouro Preto	Minas Gerais	20°23'S 43°30'W	January 2000	IBSP 7640
A. sculptum		1	*C. prehensilis*	Santa Helena de Minas	Minas Gerais	16°58'S 40°41'W	December 1952	IBSP 4596
A. sculptum		1	*C. blumenbachii*	São João del-Rei	Minas Gerais	21°8'S 44°15'W	December 1937	IBSP 1212
A. sculptum		1	unknown	São João del-Rei	Minas Gerais	21°8'S 44°15'W	January 1940	IBSP 1862
A. sculptum		1	*F. catus*	São João del-Rei	Minas Gerais	21°8'S 44°15'W	December 1937	IBSP 1213
A. sculptum	1	1	*H. hydrochaeris*	São João del-Rei	Minas Gerais	21°8'S 44°15'W	November 1937	IBSP 1206
A. sculptum	7	1	*H. hydrochaeris*	São João del-Rei	Minas Gerais	21°8'S 44°15'W	March 1939	IBSP 1660
A. sculptum	3	6	*T. terrestris*	São João del-Rei	Minas Gerais	21°8'S 44°15'W	November 1937	IBSP 1209
A. sculptum		5	*T. terrestris*	São João del-Rei	Minas Gerais	21°8'S 44°15'W	March 1939	IBSP 1659
A. sculptum		2	*T. terrestris*	São João del-Rei	Minas Gerais	21°8'S 44°15'W	March 1939	IBSP 1668
A. sculptum	3	1	*H. sapiens*	Resende	Rio de Janeiro	22°28'S 44°26'W	September 1950	IBSP 4439

A. sculptum		1	free living	Resende	Rio de Janeiro	22°28’S 44°26’W	April 1995	IBSP 7587
A. sculptum	1	1	unknown	Rio de Janeiro	Rio de Janeiro	22°54’S 43°12’W	November 1938	IBSP 1534
A. sculptum		2	*E. caballus*	Seropédica	Rio de Janeiro	22°44’S 43°42’W	October 2000	IBSP 7107
A. sculptum	1	1	unknown	Amparo	São Paulo	22°42’S 46°45’W	March 2004	IBSP 8941
A. sculptum		4	unknown	Atibaia	São Paulo	23°7’S 46°33’W	November 1953	IBSP 4944
A. sculptum	10	13	*E. caballus*	Atibaia	São Paulo	23°7’S 46°33’W	December 1952	IBSP 4594
A. sculptum		1	*H. sapiens*	Atibaia	São Paulo	23°7’S 46°33’W	October 2001	IBSP 7451
A. sculptum	5	5	*O. aries*	Atibaia	São Paulo	23°7’S 46°33’W	December 1952	IBSP 4595
A. sculptum	3	1	*R. rufescens*	Avaré	São Paulo	23°5’S 48°55’W	August 1934	IBSP 797
A. sculptum		1	unknown	Barueri	São Paulo	23°30’S 46°52’W	June 2006	IBSP 9753
A. sculptum		1	unknown	Barueri	São Paulo	23°30’S 46°52’W	September 2007	IBSP 10444
A. sculptum	1	1	unknown	Barueri	São Paulo	23°30’S 46°52’W	March 2012	IBSP 10599
A. sculptum	20	4	unknown	Bauru	São Paulo	22°18’S 49°3’W	February 1942	IBSP 4283
A. sculptum		2	*P. concolor*	Bebedouro	São Paulo	20°56’S 48°28’W	May 1952	IBSP 4521
A. sculptum		3	unknown	Botucatu	São Paulo	22°53’S 48°26’W	February 1942	IBSP 4418
A. sculptum	1	1	*H. hydrochaeris*	Botucatu	São Paulo	22°53’S 48°26’W	September 1997	IBSP 6892
A. sculptum	1	3	*H. hydrochaeris*	Botucatu	São Paulo	22°53’S 48°26’W	September 1997	IBSP 6896
A. sculptum	1	3	*H. hydrochaeris*	Botucatu	São Paulo	22°53’S 48°26’W	September 1997	IBSP 6899
A. sculptum		1	*E. caballus*	Caçapava	São Paulo	23°6’S 45°42’W	March 2012	IBSP 10602
A. sculptum		1	*H. sapiens*	Carapicuíba	São Paulo	23°31’S 46°50’W	November 2005	IBSP 9551
A. sculptum	12	12	unknown	Casa Branca	São Paulo	21°46’S 47°5’W	January 1937	IBSP 1035
A. sculptum		1	*H. sapiens*	Catanduva	São Paulo	21°8’S 48°58’W	December 2005	IBSP 9611
A. sculptum		1	free living	Charqueada	São Paulo	22°30’S 47°46’W	May 1995	IBSP 6941
A. sculptum		1	unknown	Cotia	São Paulo	23°36’S 46°55’W	April 2007	IBSP 9811
A. sculptum		1	*E. sexcinctus*	Cotia	São Paulo	23°36’S 46°55’W	January 1973	IBSP 9253
A. sculptum		6	*Holochilus sp.*	Cotia	São Paulo	23°36’S 46°55’W	June 2012	IBSP 11084
A. sculptum	1	2	*M. gouazoubira*	Cotia	São Paulo	23°36’S 46°55’W	October 1934	IBSP 839
A. sculptum		1	*H. sapiens*	Embu-Guaçu	São Paulo	23°49’S 46°48’W	January 2010	IBSP 10374
A. sculptum		1	*H. sapiens*	Francisco Morato	São Paulo	23°16’S 46°44’W	November 2005	IBSP 9548
A. sculptum		1	*H. sapiens*	Francisco Morato	São Paulo	23°16’S 46°44’W	November 2005	IBSP 9549
A. sculptum		1	*H. sapiens*	Francisco Morato	São Paulo	23°16’S 46°44’W	December 2005	IBSP 9594
A. sculptum	1	3	unknown	Gália	São Paulo	22°17’S 49°33’W	October 1933	IBSP 1289
A. sculptum	4	4	unknown	Guarulhos	São Paulo	23°27’S 46°31’W	October 2013	IBSP 11573
A. sculptum		2	*B. taurus*	Herculândia	São Paulo	22°0’S 50°23’W	October 1952	IBSP 4577
A. sculptum		4	*H. sapiens*	Ibiúna	São Paulo	23°39’S 47°13’W	December 2005	IBSP 9606
A. sculptum		1	unknown	Iporanga	São Paulo	24°35’S 48°35’W	March 2006	IBSP 9734
A. sculptum		1	*H. sapiens*	Itapetininga	São Paulo	23°35’S 48°3’W	February 2006	IBSP 9625
A. sculptum	1	2	*C. familiaris*	Itapevi	São Paulo	23°32’S 46°56’W	January 1999	IBSP 6994
A. sculptum		1	unknown	Itapevi	São Paulo	23°32’S 46°56’W	November 1999	IBSP 7176

A. sculptum		1	*D. aurita*	Itapevi	São Paulo	23°32’S 46°56’W	November 1999	IBSP 7235
A. sculptum		1	free living	Itapevi	São Paulo	23°32’S 46°56’W	January 2000	IBSP 7073
A. sculptum	9	13	*M. americana*	Itapura	São Paulo	20°38’S 51°30’W	May 1950	IBSP 4448
A. sculptum		1	unknown	Itupeva	São Paulo	23°9’S 47°3’W	January 2009	IBSP 10470
A. sculptum	6	20	unknown	Jundiaí	São Paulo	23°11’S 46°53’W	February 2006	IBSP 9624
A. sculptum	98	2	*H. hydrochaeris*	Lins	São Paulo	21°40’S 49°44’W	March 1937	IBSP 1251
A. sculptum	5	4	*H. hydrochaeris*	Lins	São Paulo	21°40’S 49°44’W	November 1937	IBSP 1392
A. sculptum	3	1	unknown	Lorena	São Paulo	22°43’S 45°7’W	April 1937	IBSP 1087
A. sculptum	3	3	*M. gouazoubira*	Mairiporã	São Paulo	23°19’S 46°35’W	August 2005	IBSP 9639
A. sculptum		1	unknown	Mogi das Cruzes	São Paulo	23°31’S 46°11’W	January 1940	IBSP 10382
A. sculptum		1	free living	Mogi das Cruzes	São Paulo	23°31’S 46°11’W	January 2011	IBSP 1871
A. sculptum		1	*C. familiaris*	Monte Alegre do Sul	São Paulo	22°40’S 46°40’W	June 2002	IBSP 8915
A. sculptum		1	*H. sapiens*	Osasco	São Paulo	23°31’S 46°47’W	May 2001	IBSP 7302
A. sculptum	1	1	*T. terrestris*	Penápolis	São Paulo	21°25’S 50°4’W	January 1934	IBSP 1412
A. sculptum		10	*T. terrestris*	Penápolis	São Paulo	21°25’S 50°4’W	January 1934	IBSP 1725
A. sculptum	28	31	*T. terrestris*	Penápolis	São Paulo	21°25’S 50°4’W	January 1934	IBSP 1726
A. sculptum		1	*C. familiaris*	Pindamonhangaba	São Paulo	22°55’S 45°27’W	December 1934	IBSP 840
A. sculptum		1	free living	Pindamonhangaba	São Paulo	22°55’S 45°27’W	May 1998	IBSP 6940
A. sculptum		21	*H. hydrochaeris*	Piracicaba	São Paulo	22°43’S 47°38’W	December 2005	IBSP 9885
A. sculptum		30	*H. hydrochaeris*	Piracicaba	São Paulo	22°43’S 47°38’W	December 2005	IBSP 9889
A. sculptum		1	unknown	Presidente Epitácio	São Paulo	21°45’S 52°6’W	January 1999	IBSP 7002
A. sculptum	1	1	*S. scrofa*	Ribeirão Pires	São Paulo	23°42’S 46°24’W	November 1939	IBSP 1801
A. sculptum	3	6	unknown	Rifaina	São Paulo	20°4’S 47°25’W	December 1937	IBSP 1216
A. sculptum	5	1	*P. tajacu*	Rosana	São Paulo	22°34’S 53°3’W	November 1947	IBSP 4417
A. sculptum		1	unknown	Santa Isabel	São Paulo	23°18’S 46°13’W	August 2007	IBSP 10425
A. sculptum	1	1	unknown	Santana de Parnaíba	São Paulo	23°26’S 46°55’W	November 2012	IBSP 11212
A. sculptum		3	free living	Santa Rita do Passa Quatro	São Paulo	21°42’S 47°28’W	February 2002	IBSP 7486
A. sculptum	2	5	*B. taurus*	São José do Barreiro	São Paulo	22°38’S 44°34’W	December 2005	IBSP 9506
A. sculptum		1	*B. taurus*	São José do Barreiro	São Paulo	22°38’S 44°34’W	December 2005	IBSP 9509
A. sculptum		6	*E. caballus*	São José do Barreiro	São Paulo	22°38’S 44°34’W	December 2005	IBSP 9508
A. sculptum		2	*E. caballus*	São José do Barreiro	São Paulo	22°38’S 44°34’W	December 2005	IBSP 9510
A. sculptum	4	5	*E. caballus*	São José do Barreiro	São Paulo	22°38’S 44°34’W	February 2007	IBSP 9843
A. sculptum		1	unknown	São Lourenço da Serra	São Paulo	23°51’S 46°56’W	March 2009	IBSP 10023
A. sculptum	1	1	*E. caballus*	São Lourenço da Serra	São Paulo	23°51’S 46°56’W	November 2005	IBSP 9651
A. sculptum		3	*H. sapiens*	São Lourenço da Serra	São Paulo	23°51’S 46°56’W	November 2005	IBSP 9577
A. sculptum	1	2	*H. sapiens*	São Lourenço da Serra	São Paulo	23°51’S 46°56’W	November 2005	IBSP 9578
A. sculptum	1	1	*H. sapiens*	São Lourenço da Serra	São Paulo	23°51’S 46°56’W	November 2005	IBSP 9579
A. sculptum		1	*A. guariba*	São Paulo	São Paulo	23°32’S 46°38’W	December 2006	IBSP 9796
A. sculptum		1	*B. taurus*	São Paulo	São Paulo	23°32’S 46°38’W	March 1932	IBSP 687

A. sculptum	1	1	*B. taurus*	São Paulo	São Paulo	23°32'S 46°38'W	November 1936	IBSP 943
A. sculptum	1	2	*B. taurus*	São Paulo	São Paulo	23°32'S 46°38'W	November 1936	IBSP 945
A. sculptum		1	*B. variegatus*	São Paulo	São Paulo	23°32'S 46°38'W	August 1935	IBSP 800
A. sculptum		1	*H. meridionalis*	São Paulo	São Paulo	23°32'S 46°38'W	December 2006	IBSP 9792
A. sculptum	2	2	*C. familiaris*	São Paulo	São Paulo	23°32'S 46°38'W	October 1934	IBSP 841
A. sculptum		1	*C. thous*	São Paulo	São Paulo	23°32'S 46°38'W	December 1997	IBSP 6937
A. sculptum	1	13	"owl"	São Paulo	São Paulo	23°32'S 46°38'W	April 2002	IBSP 8399
A. sculptum		1	unknown	São Paulo	São Paulo	23°32'S 46°38'W	May 1936	IBSP 770
A. sculptum		1	unknown	São Paulo	São Paulo	23°32'S 46°38'W	February 1942	IBSP 4277
A. sculptum		1	unknown	São Paulo	São Paulo	23°32'S 46°38'W	March 2006	IBSP 9736
A. sculptum		1	unknown	São Paulo	São Paulo	23°32'S 46°38'W	April 2006	IBSP 9740
A. sculptum		1	unknown	São Paulo	São Paulo	23°32'S 46°38'W	April 2006	IBSP 9741
A. sculptum		2	unknown	São Paulo	São Paulo	23°32'S 46°38'W	October 2006	IBSP 9782
A. sculptum		1	unknown	São Paulo	São Paulo	23°32'S 46°38'W	July 2006	IBSP 9825
A. sculptum		1	unknown	São Paulo	São Paulo	23°32'S 46°38'W	December 1937	IBSP 1218
A. sculptum	1	3	unknown	São Paulo	São Paulo	23°32'S 46°38'W	January 2010	IBSP 10377
A. sculptum		1	unknown	São Paulo	São Paulo	23°32'S 46°38'W	August 2007	IBSP 10467
A. sculptum		1	*D. albiventris*	São Paulo	São Paulo	23°32'S 46°38'W	October 2006	IBSP 9796
A. sculptum	3	1	*E. caballus*	São Paulo	São Paulo	23°32'S 46°38'W	August 1936	IBSP 683
A. sculptum		1	*E. caballus*	São Paulo	São Paulo	23°32'S 46°38'W	December 1934	IBSP 771
A. sculptum	3	2	*E. caballus*	São Paulo	São Paulo	23°32'S 46°38'W	October 1934	IBSP 844
A. sculptum	1	1	*E. caballus*	São Paulo	São Paulo	23°32'S 46°38'W	December 1930	IBSP 1277
A. sculptum	2	3	*E. caballus*	São Paulo	São Paulo	23°32'S 46°38'W	December 2007	IBSP 9968
A. sculptum		1	*H. sapiens*	São Paulo	São Paulo	23°32'S 46°38'W	January 1940	IBSP 1825
A. sculptum		1	*H. sapiens*	São Paulo	São Paulo	23°32'S 46°38'W	November 2005	IBSP 9555
A. sculptum		1	*H. sapiens*	São Paulo	São Paulo	23°32'S 46°38'W	November 2005	IBSP 9571
A. sculptum		1	*H. sapiens*	São Paulo	São Paulo	23°32'S 46°38'W	January 2006	IBSP 9616
A. sculptum		1	*H. sapiens*	São Paulo	São Paulo	23°32'S 46°38'W	January 2006	IBSP 9617
A. sculptum		1	*H. sapiens*	São Paulo	São Paulo	23°32'S 46°38'W	January 2006	IBSP 9618
A. sculptum		1	*H. sapiens*	São Paulo	São Paulo	23°32'S 46°38'W	January 2006	IBSP 9619
A. sculptum		1	*H. hydrochaeris*	São Paulo	São Paulo	23°32'S 46°38'W	March 2004	IBSP 8940
A. sculptum		1	*H. hydrochaeris*	São Paulo	São Paulo	23°32'S 46°38'W	December 2003	IBSP 8912
A. sculptum	1	1	*H. hydrochaeris*	São Paulo	São Paulo	23°32'S 46°38'W	October 2002	IBSP 7989
A. sculptum	5	7	*H. hydrochaeris*	São Paulo	São Paulo	23°32'S 46°38'W	June 2002	IBSP 7760
A. sculptum		2	*H. hydrochaeris*	São Paulo	São Paulo	23°32'S 46°38'W	February 2002	IBSP 7562
A. sculptum		1	*H. hydrochaeris*	São Paulo	São Paulo	23°32'S 46°38'W	February 2002	IBSP 7484
A. sculptum	1	3	*H. hydrochaeris*	São Paulo	São Paulo	23°32'S 46°38'W	January 2002	IBSP 7459
A. sculptum	2	2	*H. hydrochaeris*	São Paulo	São Paulo	23°32'S 46°38'W	October 2001	IBSP 7444
A. sculptum		3	*H. hydrochaeris*	São Paulo	São Paulo	23°32'S 46°38'W	October 2001	IBSP 7440

A. sculptum		4	*H. hydrochaeris*	São Paulo	São Paulo	23°32'S 46°38'W	September 2000	IBSP 7049
A. sculptum	1	1	*M. gouazoubira*	São Paulo	São Paulo	23°32'S 46°38'W	August 2006	IBSP 9775
A. sculptum		1	*M. tridactyla*	São Paulo	São Paulo	23°32'S 46°38'W	January 1940	IBSP 1861
A. sculptum		1	*S. villosus*	São Paulo	São Paulo	23°32'S 46°38'W	September 2002	IBSP 7967
A. sculptum	1	1	*S. scrofa*	São Paulo	São Paulo	23°32'S 46°38'W	May 1939	IBSP 1717
A. sculptum		1	free living	São Paulo	São Paulo	23°32'S 46°38'W	April 1938	IBSP 1307
A. sculptum	2	5	free living	São Paulo	São Paulo	23°32'S 46°38'W	January 1940	IBSP 1827
A. sculptum		2	free living	São Paulo	São Paulo	23°32'S 46°38'W	February 1940	IBSP 1874
A. sculptum	3	1	free living	São Paulo	São Paulo	23°32'S 46°38'W	December 2000	IBSP 7060
A. sculptum		1	free living	São Paulo	São Paulo	23°32'S 46°38'W	October 2000	IBSP 7061
A. sculptum	4	13	free living	São Paulo	São Paulo	23°32'S 46°38'W	November 2000	IBSP 7169
A. sculptum	1	1	free living	São Paulo	São Paulo	23°32'S 46°38'W	April 2001	IBSP 7286
A. sculptum		1	free living	São Paulo	São Paulo	23°32'S 46°38'W	May 2001	IBSP 7305
A. sculptum		1	free living	São Paulo	São Paulo	23°32'S 46°38'W	November 2000	IBSP 7429
A. sculptum		2	unknown	São Roque	São Paulo	23°31'S 47°8'W	March 2000	IBSP 7209
A. sculptum		1	unknown	São Roque	São Paulo	23°31'S 47°8'W	April 2000	IBSP 7211
A. sculptum	1	2	unknown	São Roque	São Paulo	23°31'S 47°8'W	April 2000	IBSP 7217
A. sculptum		1	*B. variegatus*	Sorocaba	São Paulo	23°30'S 47°27'W	September 1934	IBSP 843
A. sculptum		1	*H. sapiens*	Taubaté	São Paulo	23°1'S 45°33'W	November 2007	IBSP 9783
A. sculptum	1	1	*H. sapiens*	Teodoro Sampaio	São Paulo	22°31'S 52°10'W	April 1951	IBSP 4463
A. sculptum	16	29	*H. sapiens*	Ubatuba	São Paulo	23°26'S 45°4'W	November 2005	IBSP 9564
A. sculptum		1	*H. sapiens*	Ubatuba	São Paulo	23°26'S 45°4'W	November 2005	IBSP 9565
A. sculptum		1	*H. sapiens*	Ubatuba	São Paulo	23°26'S 45°4'W	November 2005	IBSP 9584
A. sculptum	1	2	*H. sapiens*	Ubatuba	São Paulo	23°26'S 45°4'W	December 2005	IBSP 9601
A. sculptum		1	*C. familiaris*	Valinhos	São Paulo	22°58'S 46°59'W	October 2002	IBSP 7990
A. sculptum		1	free living	Londrina	Paraná	23°18'S 51°9'W	March 2005	IBSP 10483
A. sculptum	6	6	*H. sapiens*	Reserva do Cabaçal	Mato Grosso	15°4'S 58°27'W	March 2002	IBSP 7647
A. sculptum	2	1	free living	Corumbá	Mato Grosso do Sul	19°0'S 57°39'W	June 1998	IBSP 6919
A. sculptum		1	free living	Corumbá	Mato Grosso do Sul	19°0'S 57°39'W	November 1998	IBSP 7416
A. sculptum	5	4	*T. terrestris*	Aimorés	Minas Gerais	19°29'S 41°3'W	August 1950	IBSP 4436
A. sculptum *		1	*D. azarae*	Miranda	Mato Grosso do Sul	20°14'S 56°22'W	August 1952	IBSP 4542
A. sculptum *		1	*T. tetradactyla*	Miranda	Mato Grosso do Sul	20°14'S 56°22'W	September 1952	IBSP 4581
A. sculptum *	9	7	*D. aurita*	Além Paraíba	Minas Gerais	21°53'S 42°42'W	October 1955	IBSP 3843
A. cajennense s. l.	1		free living	Ilhéus	Bahia	14°47'S 39°2'W	January 1999	IBSP 7615
A. cajennense s. l.	1		free living	Jussari	Bahia	15°11'S 39°29'W	April 1998	IBSP 7043
A. cajennense s. l.	3		free living	Bonito	Mato Grosso do Sul	21°7'S 56°28'W	October 2002	IBSP 8933
A. cajennense s. l.	1		free living	Corumbá	Mato Grosso do Sul	19°0'S 57°39'W	January 2000	IBSP 7635
A. cajennense s. l.	1		free living	Corumbá	Mato Grosso do Sul	19°0'S 57°39'W	August 2005	IBSP 9513
A. cajennense s. l.	4		*P. tajacu*	Araguari	Minas Gerais	18°38'S 48°11'W	October 1951	IBSP 4494

A. cajennense s. l.	1		unknown	Matozinhos	Minas Gerais	19°33′S 44°4′W	March 2013	IBSP 11602
A. cajennense s. l.	3		*C. familiaris*	Álvares Machado	São Paulo	22°4′S 51°28′W	October 1951	IBSP 4486
A. cajennense s. l.	1		unknown	Igaratá	São Paulo	23°12′S 46°9′W	November 2007	IBSP 9788
A. cajennense s. l.	2		*C. familiaris*	Itatiba	São Paulo	23°0′S 46°50′W	July 2011	IBSP 10557
A. cajennense s. l.	1		unknown	Itatiba	São Paulo	23°0′S 46°50′W	April 2007	IBSP 9824
A. cajennense s. l.	1		unknown	Itirapina	São Paulo	22°15′S 47°49′W	December 2000	IBSP 9056
A. cajennense s. l.	1		unknown	Itú	São Paulo	23°15′S 47°17′W	May 2005	IBSP 10035
A. cajennense s. l.	1		unknown	Ipueiras	Tocantins	11°14′S 48°27′W	September 2001	IBSP 7607
A. cajennense s. l.	5		*S. scrofa*	Macapá	Amapá	0°2′N 51°3′W	March 1983	IBSP 7101
A. cajennense s. l.	1		unknown	Manaus	Amazonas	3°6′S 60°1′W	May 1955	IBSP 5732
A. cajennense s. l.	1		free living	Canaã dos Carajás	Pará	6°29′S 49°52′W	December 1970	IBSP 6861
A. cajennense s. l.	1		*H. sapiens*	Monte Alegre	Pará	2°0′S 54°4′W	October 2007	IBSP 9895
A. cajennense s. l.	2		*Bradypus sp*	Tucuruí	Pará	3°46′S 49°40′W	July 1984	IBSP 6775
A. cajennense s. l.	1		unknown	Tucuruí	Pará	3°46′S 49°40′W	July 1984	IBSP 6773
A. cajennense s. l.	1		*H. sapiens*	Tucuruí	Pará	3°46′S 49°40′W	July 1984	IBSP 6755
A. cajennense s. l.	1		*H. sapiens*	Tucuruí	Pará	3°46′S 49°40′W	July 1984	IBSP 6767
A. cajennense s. l.	1		free living	Ilhéus	Bahia	14°47′S 39°2′W	January 1999	IBSP 7612
A. cajennense s. l.	3		*M. tridactyla*	Bataguassu	Mato Grosso do Sul	21°42′S 52°25′W	April 2001	IBSP 7284
A. cajennense s. l.	1		unknown	Campo Grande	Mato Grosso do Sul	20°26′S 54°38′W	July 1995	IBSP 7585
A. cajennense s. l.	1		unknown	Corumbá	Mato Grosso do Sul	19°0′S 57°39′W	May 1999	IBSP 7419
A. cajennense s. l.	1		*H. sapiens*	Corumbá	Mato Grosso do Sul	19°0′S 57°39′W	August 2005	IBSP 9515
A. cajennense s. l.	1		free living	Corumbá	Mato Grosso do Sul	19°0′S 57°39′W	January 2000	IBSP 7631
A. cajennense s. l.	1		free living	Corumbá	Mato Grosso do Sul	19°0′S 57°39′W	January 2000	IBSP 7632
A. cajennense s. l.	1		free living	Corumbá	Mato Grosso do Sul	19°0′S 57°39′W	January 2000	IBSP 7633
A. cajennense s. l.	1		free living	Ivinhema	Mato Grosso do Sul	22°18′S 53°48′W	January 2004	IBSP 9351
A. cajennense s. l.	18		*M. tridactyla*	Maracaju	Mato Grosso do Sul	21°36′S 55°10′W	October 1952	IBSP 4563
A. cajennense s. l.	1		*T. tetradactyla*	Miranda	Mato Grosso do Sul	20°14′S 56°22′W	September 1952	IBSP 4570
A. cajennense s. l.	14		*M. tridactyla*	Ribas do Rio Pardo	Mato Grosso do Sul	20°26′S 53°45′W	October 1952	IBSP 4546
A. cajennense s. l.	5		*M. tridactyla*	Ribas do Rio Pardo	Mato Grosso do Sul	20°26′S 53°45′W	September 1952	IBSP 4578
A. cajennense s. l.	1		free living	Santa Rita do Pardo	Mato Grosso do Sul	21°18′S 52°49′W	April 2001	IBSP 7479
A. cajennense s. l.	2		*T. terrestris*	Três Lagoas	Mato Grosso do Sul	20°45′S 51°40′W	January 1952	IBSP 4509
A. cajennense s. l.	1		*H. sapiens*	Juatuba	Minas Gerais	19°57′S 44°20′W	April 2002	IBSP 7649
A. cajennense s. l.	4		*H. hydrochaeris*	São João del-Rei	Minas Gerais	21°8′S 44°15′W	May 1938	IBSP 1656
A. cajennense s. l.	2		*T. terrestris*	São Romão	Minas Gerais	16°22′S 45°4′W	April 1939	IBSP 1893
A. cajennense s. l.	1		unknown	Paraty	Rio de Janeiro	23°13′S 44°42′W	August 2006	IBSP 9755
A. cajennense s. l.	1		*E. caballus*	Rio de Janeiro	Rio de Janeiro	22°54′S 43°12′W	January 1930	IBSP 842
A. cajennense s. l.	1		*E. caballus*	Seropédica	Rio de Janeiro	22°44′S 43°42′W	June 2003	IBSP 8748
A. cajennense s. l.	1		*E. caballus*	Seropédica	Rio de Janeiro	22°44′S 43°42′W	June 2003	IBSP 8751
A. cajennense s. l.	23		*E. caballus*	Araçariguama	São Paulo	23°26′S 47°3′W	November 2007	IBSP 10056

A. cajennense s. l.	1		unknown	Araraquara	São Paulo	21°47′S 48°10′W	January 2010	IBSP 10379
A. cajennense s. l.	1		*S. nigritus*	Bebedouro	São Paulo	20°56′S 48°28′W	October 1950	IBSP 4451
A. cajennense s. l.	1		*H. hydrochaeris*	Botucatu	São Paulo	22°53′S 48°26′W	September 1997	IBSP 6897
A. cajennense s. l.	2		*H. hydrochaeris*	Botucatu	São Paulo	22°53′S 48°26′W	September 1997	IBSP 6898
A. cajennense s. l.	1		*H. hydrochaeris*	Botucatu	São Paulo	22°53′S 48°26′W	September 1997	IBSP 6900
A. cajennense s. l.	3		*H. hydrochaeris*	Botucatu	São Paulo	22°53′S 48°26′W	September 1997	IBSP 6901
A. cajennense s. l.	1		unknown	Bragança Paulista	São Paulo	22°57′S 46°32′W	November 2006	IBSP 9791
A. cajennense s. l.	1		*H. sapiens*	Bragança Paulista	São Paulo	22°57′S 46°32′W	November 2005	IBSP 9572
A. cajennense s. l.	1		*H. sapiens*	Caieiras	São Paulo	23°21′S 46°44′W	November 2005	IBSP 9588
A. cajennense s. l.	1		unknown	Carapicuíba	São Paulo	23°31′S 46°50′W	May 2013	IBSP 11245
A. cajennense s. l.	61		*E. caballus*	Colina	São Paulo	20°42′S 48°32′W	October 1957	IBSP 5177
A. cajennense s. l.	1		*M. tridactyla*	Conchas	São Paulo	23°0′S 48°0′W	October 1952	IBSP 4560
A. cajennense s. l.	1		unknown	Embu-Guaçu	São Paulo	23°49′S 46°48′W	December 2012	IBSP 11122
A. cajennense s. l.	2		unknown	Gália	São Paulo	22°17′S 49°33′W	October 1933	IBSP 1290
A. cajennense s. l.	1		free living	Guararema	São Paulo	23°24′S 46°2′W	February 2002	IBSP 7482
A. cajennense s. l.	1		free living	Guarulhos	São Paulo	23°27′S 46°31′W	September 2001	IBSP 7427
A. cajennense s. l.	1		*E. caballus*	Itapevi	São Paulo	23°32′S 46°56′W	October 2000	IBSP 7216
A. cajennense s. l.	1		free living	Itapevi	São Paulo	23°32′S 46°56′W	December 1999	IBSP 7178
A. cajennense s. l.	2		*H. hydrochaeris*	Lins	São Paulo	21°40′S 49°44′W	December 1937	IBSP 1227
A. cajennense s. l.	1		*E. caballus*	Mogi Guaçu	São Paulo	22°22′S 46°56′W	January 2010	IBSP 10380
A. cajennense s. l.	1		*T. terrestris*	Penápolis	São Paulo	21°25′S 50°4′W	January 1934	IBSP 1724
A. cajennense s. l.	1		free living	Pindamonhangaba	São Paulo	22°55′S 45°27′W	September 1995	IBSP 6952
A. cajennense s. l.	29		*H. hydrochaeris*	Piracicaba	São Paulo	22°43′S 47°38′W	December 2005	IBSP 9881
A. cajennense s. l.	30		*H. hydrochaeris*	Piracicaba	São Paulo	22°43′S 47°38′W	December 2005	IBSP 9882
A. cajennense s. l.	30		*H. hydrochaeris*	Piracicaba	São Paulo	22°43′S 47°38′W	December 2005	IBSP 9884
A. cajennense s. l.	25		*H. hydrochaeris*	Piracicaba	São Paulo	22°43′S 47°38′W	December 2005	IBSP 9886
A. cajennense s. l.	60		*H. hydrochaeris*	Piracicaba	São Paulo	22°43′S 47°38′W	December 2005	IBSP 9888
A. cajennense s. l.	1		unknown	Santa Isabel	São Paulo	23°18′S 46°13′W	March 2008	IBSP 10418
A. cajennense s. l.	1		free living	Santa Isabel	São Paulo	23°18′S 46°13′W	October 1996	IBSP 6951
A. cajennense s. l.	1		free living	Santa Isabel	São Paulo	23°18′S 46°13′W	October 1996	IBSP 6954
A. cajennense s. l.	1		free living	Santa Isabel	São Paulo	23°18′S 46°13′W	October 1996	IBSP 6955
A. cajennense s. l.	1		unknown	Santo André	São Paulo	23°39′S 46°32′W	November 2006	IBSP 9790
A. cajennense s. l.	1		*M. tridactyla*	São Bernardo do Campo	São Paulo	23°41′S 46°33′W	December 1999	IBSP 7164
A. cajennense s. l.	1		free living	São José dos Campos	São Paulo	23°10′S 45°53′W	December 2007	IBSP 9967
A. cajennense s. l.	1		*H. sapiens*	São Lourenço da Serra	São Paulo	23°51′S 46°56′W	November 2005	IBSP 9586
A. cajennense s. l.	1		*H. sapiens*	São Lourenço da Serra	São Paulo	23°51′S 46°56′W	November 2005	IBSP 9593
A. cajennense s. l.	1		unknown	São Luiz do Paraitinga	São Paulo	23°13′S 45°18′W	March 2007	IBSP 9805
A. cajennense s. l.	1		unknown	São Luiz do Paraitinga	São Paulo	23°13′S 45°18′W	March 2006	IBSP 9732
A. cajennense s. l.	2		*B. taurus*	São Paulo	São Paulo	23°32′S 46°38′W	November 1936	IBSP 958

A. cajennense s. l.	1		*C. thous*	São Paulo	São Paulo	23°32’S 46°38’W	November 1934	IBSP 838
A. cajennense s. l.	1		*C. thous*	São Paulo	São Paulo	23°32’S 46°38’W	December 1999	IBSP 7157
A. cajennense s. l.	1		unknown	São Paulo	São Paulo	23°32’S 46°38’W	April 2006	IBSP 9743
A. cajennense s. l.	1		unknown	São Paulo	São Paulo	23°32’S 46°38’W	August 2007	IBSP 9846
A. cajennense s. l.	1		unknown	São Paulo	São Paulo	23°32’S 46°38’W	July 2013	IBSP 11286
A. cajennense s. l.	1		*E. caballus*	São Paulo	São Paulo	23°32’S 46°38’W	June 2003	IBSP 8748
A. cajennense s. l.	1		*E. caballus*	São Paulo	São Paulo	23°32’S 46°38’W	June 2003	IBSP 8751
A. cajennense s. l.	2		*H. sapiens*	São Paulo	São Paulo	23°32’S 46°38’W	March 1932	IBSP 685
A. cajennense s. l.	1		*H. sapiens*	São Paulo	São Paulo	23°32’S 46°38’W	March 1937	IBSP 1082
A. cajennense s. l.	1		*H. sapiens*	São Paulo	São Paulo	23°32’S 46°38’W	August 1940	IBSP 1992
A. cajennense s. l.	1		*H. sapiens*	São Paulo	São Paulo	23°32’S 46°38’W	November 2005	IBSP 9566
A. cajennense s. l.	1		*H. sapiens*	São Paulo	São Paulo	23°32’S 46°38’W	November 2005	IBSP 9580
A. cajennense s. l.	1		*H. sapiens*	São Paulo	São Paulo	23°32’S 46°38’W	December 2005	IBSP 9599
A. cajennense s. l.	1		*H. sapiens*	São Paulo	São Paulo	23°32’S 46°38’W	December 2005	IBSP 9608
A. cajennense s. l.	1		*H. sapiens*	São Paulo	São Paulo	23°32’S 46°38’W	February 2006	IBSP 9623
A. cajennense s. l.	1		*H. sapiens*	São Paulo	São Paulo	23°32’S 46°38’W	February 2006	IBSP 9626
A. cajennense s. l.	6		*H. hydrochaeris*	São Paulo	São Paulo	23°32’S 46°38’W	March 1939	IBSP 1776
A. cajennense s. l.	1		*H. hydrochaeris*	São Paulo	São Paulo	23°32’S 46°38’W	June 1937	IBSP 1101
A. cajennense s. l.	9		*H. hydrochaeris*	São Paulo	São Paulo	23°32’S 46°38’W	December 1997	IBSP 6938
A. cajennense s. l.	1		*M. tridactyla*	São Paulo	São Paulo	23°32’S 46°38’W	October 1952	IBSP 4572
A. cajennense s. l.	1		*R. magnirostris*	São Paulo	São Paulo	23°32’S 46°38’W	October 2004	IBSP 9650
A. cajennense s. l.	1		*H. sapiens*	Vargem Grande Paulista	São Paulo	23°36’S 47°1’W	November 2005	IBSP 9592
A. cajennense s. l.	1		*C. thous*	Japurá	Paraná	23°28’S 52°33’W	December 1952	IBSP 4599
A. cajennense s. l.	2		unknown	São José dos Pinhais	Paraná	25°32’S 49°12’W	April 1937	IBSP 1089
A. cajennense s. l.	1		*C. familiaris*	Presidente Getúlio	Santa Catarina	27°3’S 49°37’W	August 1936	IBSP 499
A. cajennense s. l.	1		unknown	Parauapebas	Pará	6°4’S 49°54’W	September 2012	IBSP 11600
A. cajennense s. l.	1		*H. sapiens*	Santana do Riacho	Minas Gerais	19°10’S 43°42’W	September 2006	IBSP 9848
A. cajennense s. l.	6		*E. caballus*	Araçariguama	São Paulo	23°26’S 47°3’W	November 2007	IBSP 10055
A. cajennense s. l.	1		unknown	Osasco	São Paulo	23°31’S 46°47’W	April 2006	IBSP 9739
A. cajennense s. l.	1		unknown	Ourinhos	São Paulo	22°58’S 49°52’W	October 1999	IBSP 7153
**Tick species**	**No. Males ****	**No. Females**	**host**	**municipality**	**state**	**latitude longitude**	**date**	**Acession number**
**CNC**								
A. cajennense s. s.	1	3	free living	Altamira	Pará	3°12’S 52°12’W	August 2012	CNC 2244
A. cajennense s. s.	3	2	*M. tridactyla*	Belém	Pará	1°27’S 48°30’W	September 2011	CNC 2615
A. cajennense s. s.	1	1	*C. paca*	Marabá	Pará	5°22’S 49°7’W	June 2001	CNC 499
A. cajennense s. s.	3	3	*T. terrestris*	Marabá	Pará	5°22’S 49°7’W	July 2001	CNC 502
A. cajennense s. s.	1	5	*T. terrestris*	Parauapebas	Pará	6°4’S 49°54’W	November 2003	CNC 827
A. cajennense s. s.	2	2	free living	Porto de Moz	Pará	1°44’S 52°14’W	March 2013	CNC 2416

A. cajennense s. s.	2	1	*S. caffer*	Santa Isabel do Pará	Pará	1°17'S 48°9'W	July 2009	CNC 2616
A. cajennense s. s.	3		*E. caballus*	São Domingos do Capim	Pará	1°40'S 47°46'W	October 2011	CNC 2617
A. cajennense s. s.	5	10	free living	Campo Novo de Rondônia	Rondônia	10°35'S 63°36'W	April 2001	CNC 446
A. cajennense s. s.	8	1	*E. caballus*	Governador Jorge Teixeira	Rondônia	10°31'S 62°38'W	January 2009	CNC 1410
A. cajennense s. s.	14	21	free living	Governador Jorge Teixeira	Rondônia	10°31'S 62°38'W	December 2008	CNC 1808
A. cajennense s. s.		1	*P. tajacu*	Governador Jorge Teixeira	Rondônia	10°31'S 62°38'W	November 2000	CNC 415
A. cajennense s. s.	1	4	free living	Governador Jorge Teixeira	Rondônia	10°31'S 62°38'W	June 2001	CNC 492
A. cajennense s. s.	1	1	free living	Governador Jorge Teixeira	Rondônia	10°31'S 62°38'W	October 2001	CNC 577
A. cajennense s. s.	24	26	free living	Governador Jorge Teixeira	Rondônia	10°31'S 62°38'W	December 2001	CNC 627
A. cajennense s. s.	6	4	free living	Governador Jorge Teixeira	Rondônia	10°31'S 62°38'W	April 2002	CNC 678
A. cajennense s. s.	9	3	*S. caffer*	São Francisco do Guaporé	Rondônia	12°3'S 63°34'W	September 2011	CNC 2618
A. cajennense s. s.		3	free living	Vilhena	Rondônia	12°44'S 60°8'E	March 2002	CNC 626
A. cajennense s. s.		1	*T. tetradactyla*	Aragominas	Tocantins	7°9'S 48°31'W	April 2013	CNC 2571
A. cajennense s. s.	1	1	*D. novemcinctus*	Lagoa da Confusão	Tocantins	10°47'S 49°37'W	April 2013	CNC 2570
A. cajennense s. s.		19	*M. tridactyla*	Lagoa da Confusão	Tocantins	10°47'S 49°37'W	May 2013	CNC 2573
A. cajennense s. s.	2	2	*B. taurus*	Pium	Tocantins	10°26'S 49°10'W	August 2009	CNC 1797
A. cajennense s. s.		1	*C. familiaris*	Pium	Tocantins	10°26'S 49°10'W	August 2009	CNC 1800
A. cajennense s. s.	2	3	*E. caballus*	Pium	Tocantins	10°26'S 49°10'W	August 2009	CNC 1794
A. cajennense s. s.	1	1	free living	Pium	Tocantins	10°26'S 49°10'W	August 2009	CNC 2625
A. cajennense s. s.	4	1	free living	Presidente Kennedy	Tocantins	8°32'S 48°30'W	November 2012	CNC 2340
A. cajennense s. s.	7	1	*E. asinus*	Chapadinha	Maranhão	3°44'S 43°21'W	January 2012	CNC 2635
A. cajennense s. s.	14	17	*E. caballus*	Chapadinha	Maranhão	3°44'S 43°21'W	January 2012	CNC 2636
A. cajennense s. s.	1	1	*E. caballus*	Milagres do Maranhão	Maranhão	3°34'S 42°36'W	February 2013	CNC 2637
A. cajennense s. s.	1	1	*E. caballus*	Peri Mirim	Maranhão	2°34'S 44°51'W	August 2013	CNC 2638
A. cajennense s. s.		1	*E. caballus*	São Bento	Maranhão	2°41'S 44°49'W	August 2011	CNC 2639
A. cajennense s. s.		1	*E. caballus*	São Bernardo	Maranhão	3°21'S 42°25'W	February 2013	CNC 2640
A. cajennense s. s.		1	*H. sapiens*	Urbano Santos	Maranhão	3°12'S 43°24'W	August 2011	CNC 2641
A. cajennense s. s.	2	2	*S. scofra*	Viana	Maranhão	3°13'S 45°0'W	August 2013	CNC 2642
A. cajennense s. s.	11	34	*T. terrestris*	Alta Floresta	Mato Grosso	9°52'S 56°5'W	December 2013	CNC 2582
A. cajennense s. s.	2	2	*E. caballus*	Confresa	Mato Grosso	10°38'S 51°34'W	April 2011	CNC 2645
A. cajennense s. s.	1	1	*T. terrestris*	Jauru	Mato Grosso	15°20'S 58°51'W	January 2011	CNC 2364
A. cajennense s. s.	20	13	*H. hydrochaeris*	Lucas do Rio Verde	Mato Grosso	13°3'S 55°54'W	November 2013	CNC 2544
A. cajennense s. s.		2	*P. onca*	Lucas do Rio Verde	Mato Grosso	13°3'S 55°54'W	April 2011	CNC 1924
A. cajennense s. s.		1	*E. caballus*	São Félix do Araguaia	Mato Grosso	11°37'S 50°40'W	May 2008	CNC 1124
A. cajennense s. s.	1	4	*H. hydrochaeris*	Sinop	Mato Grosso	11°50'S 55°38'W	July 2012	CNC 2222
A. cajennense s. s.	1	3	*P. tajacu*	Sinop	Mato Grosso	11°50'S 55°38'W	October 2012	CNC 2271
A. cajennense s. s.		1	*M. tridactyla*	Araguaína	Tocantins	7°11'S 48°12'W	April 2013	CNC 2574
A. cajennense s. s.		5	*B. taurus*	Caseara	Tocantins	9°16'S 49°57'W	May 2009	CNC 1707
A. cajennense s. s.		1	*B. taurus*	Goiatins	Tocantins	7°42'S 47°18'W	September 2007	CNC 1062

A. cajennense s. s.		4	*M. tridactyla*	Gurupi	Tocantins	11°43'S 49°4'W	November 2012	CNC 2328
*A. cajennense s. s.**	14	6	free living	Governador Jorge Teixeira	Rondônia	10°31'S 62°38'W	October 2002	CNC 599
*A. cajennense s. s.**		14	*C. familiaris*	Cristalândia	Tocantins	10°36'S 49°11'W	August 2011	CNC 2619
*A. cajennense s. s.**	2	1	*E. caballus*	Cristalândia	Tocantins	10°36'S 49°11'W	August 2011	CNC 2620
*A. cajennense s. s.**		1	*B. taurus*	Lagoa da Confusão	Tocantins	10°47'S 49°37'W	September 2011	CNC 2621
*A. cajennense s. s.**		2	*C. familiaris*	Lagoa da Confusão	Tocantins	10°47'S 49°37'W	September 2011	CNC 2622
*A. cajennense s. s.**		2	*B. taurus*	Pium	Tocantins	10°26'S 49°10'W	August 2011	CNC 2623
*A. cajennense s. s.**		2	*E. asinus*	Pium	Tocantins	10°26'S 49°10'W	August 2011	CNC 2624
*A. cajennense s. s.**	1	1	*C. familiaris*	Barreirinhas	Maranhão	2°44'S 42°49'W	August 2011	CNC 2632
A. sculptum		6	*M. tridactyla*	Araguaína	Tocantins	7°11'S 48°12'W	April 2013	CNC 2574
A. sculptum	1	1	*T. tetradactyla*	Araguaína	Tocantins	7°11'S 48°12'W	April 2013	CNC 2507
A. sculptum		3	*M. tridactyla*	Lagoa da Confusão	Tocantins	10°47'S 49°37'W	May 2013	CNC 2573
A. sculptum	2	12	*H. hydrochaeris*	Palmas	Tocantins	10°11'S 48°20'W	January 20013	CNC 2445
A. sculptum		1	*E. asinus*	Pium	Tocantins	10°26'S 49°10'W	August 2011	CNC 2624
A. sculptum	5	7	free living	Tocantinópolis	Tocantins	6°19'S 47°24'W	June 2011	CNC 2626
A. sculptum	5	4	*E. caballus*	Campo Formoso	Bahia	10°30'S 40°19'W	August 2012	CNC 2627
A. sculptum	12	32	*E. caballus*	Teolândia	Bahia	13°36'S 39°29'W	October 2006	CNC 2629
A. sculptum	1	1	*E. caballus*	Açailândia	Maranhão	4°56'S 47°30'W	August 2013	CNC 2630
A. sculptum	3	1	*E. caballus*	Campestre do Maranhão	Maranhão	6°10'S 47°21'W	January 2012	CNC 2633
A. sculptum	1	2	*C. familiaris*	Chapadinha	Maranhão	3°44'S 43°21'W	February 2010	CNC 2135
A. sculptum	1	1	*C. familiaris*	Chapadinha	Maranhão	3°44'S 43°21'W	March 2010	CNC 2634
A. sculptum	13	5	*E. caballus*	Amaraji	Pernambuco	8°22'S 35°27'W	November 2007	CNC 1314
A. sculptum	1	1	*H. sapiens*	Gilbués	Piauí	9°49'S 45°20'W	January 2005	CNC 889
A. sculptum	1	3	*H. sapiens*	Gilbués	Piauí	9°49'S 45°20'W	November 2009	CNC 1496
A. sculptum	5	5	*E. asinus*	José de Freitas	Piauí	4°45'S 42°34'W	January 2011	CNC 2643
A. sculptum		2	*C. familiaris*	Cumari	Goiás	18°15'S 48°9'W	April 2013	CNC 2438
A. sculptum	7	1	*E. caballus*	Cumari	Goiás	18°15'S 48°9'W	January 2012	CNC 2214
A. sculptum	6	8	*E. caballus*	Cumari	Goiás	18°15'S 48°9'W	June 2011	CNC 2644
A. sculptum	2	2	*H. sapiens*	Cumari	Goiás	18°15'S 48°9'W	February 2012	CNC 2213
A. sculptum		1	*C. brachyurus*	Mineiros	Goiás	17°34'S 52°33'W	October 1999	CNC 312
A. sculptum		1	*C. brachyurus*	Mineiros	Goiás	17°34'S 52°33'W	January 2006	CNC 1696
A. sculptum		2	*M. tridactyla*	Mineiros	Goiás	17°34'S 52°33'W	November 1999	CNC 259
A. sculptum		2	*P. onca*	Mineiros	Goiás	17°34'S 52°33'W	April 2000	CNC 313
A. sculptum	4	5	*P. onca*	Mineiros	Goiás	17°34'S 52°33'W	July 2002	CNC 767
A. sculptum	1	7	*P. onca*	Mineiros	Goiás	17°34'S 52°33'W	January 2007	CNC 1691
A. sculptum		1	*P. onca*	Mineiros	Goiás	17°34'S 52°33'W	November 2008	CNC 1699
A. sculptum	2	7	*P. tajacu*	Mineiros	Goiás	17°34'S 52°33'W	April 2002	CNC 768
A. sculptum	2	1	*P. maximus*	Mineiros	Goiás	17°34'S 52°33'W	January 2005	CNC 1681
A. sculptum		6	*T. terrestris*	Mineiros	Goiás	17°34'S 52°33'W	April 2000	CNC 315

A. sculptum	45	28	*T. terrestris*	Mineiros	Goiás	17°34'S 52°33'W	June 2002	CNC 776
A. sculptum	46	40	*T. pecari*	Mineiros	Goiás	17°34'S 52°33'W	June 2001	CNC 769
A. sculptum	58	53	*T. pecari*	Mineiros	Goiás	17°34'S 52°33'W	November 2001	CNC 770
A. sculptum	18	39	*T. pecari*	Mineiros	Goiás	17°34'S 52°33'W	January 2002	CNC 772
A. sculptum	17	8	*T. pecari*	Mineiros	Goiás	17°34'S 52°33'W	March 2003	CNC 821
A. sculptum		1	*T. pecari*	Mineiros	Goiás	17°34'S 52°33'W	July 2004	CNC 1655
A. sculptum	3	3	free living	Mineiros	Goiás	17°34'S 52°33'W	November 2000	CNC 298
A. sculptum	9	15	*C. thous*	Barão de Melgaço	Mato Grosso	16°11'S 55°58'W	September 2003	CNC 802
A. sculptum	1	3	*Puma concolor*	Barão de Melgaço	Mato Grosso	16°11'S 55°58'W	January 2002	CNC 598
A. sculptum		3	*Puma concolor*	Barão de Melgaço	Mato Grosso	16°11'S 55°58'W	January 2003	CNC 760
A. sculptum	5	4	*T. terrestris*	Chapada dos Guimarães	Mato Grosso	15°27'S 55°45'W	June 2001	CNC 554
A. sculptum		5	*M. gouazoubira*	Cuiabá	Mato Grosso	15°35'S 56°5'W	July 2012	CNC 2275
A. sculptum	1	1	*H. sapiens*	Jauru	Mato Grosso	15°20'S 58°51'W	March 2001	CNC 433
A. sculptum		7	*P. onca*	Lucas do Rio Verde	Mato Grosso	13°3'S 55°54'W	April 2011	CNC 1924
A. sculptum	1	1	*H. sapiens*	Nobres	Mato Grosso	14°43'S 56°19'W	June 2012	CNC 2646
A. sculptum	5	2	free living	Nobres	Mato Grosso	14°43'S 56°19'W	June 2012	CNC 2314
A. sculptum	1	21	free living	Nobres	Mato Grosso	14°43'S 56°19'W	June 2012	CNC 2647
A. sculptum	2	3	*S. venaticus*	Nova Xavantina	Mato Grosso	14°40'S 52°21'W	June 2012	CNC 2212
A. sculptum		1	*C. familiaris*	Poconé	Mato Grosso	16°15'S 56°37'W	April 2002	CNC 648
A. sculptum	2	12	*E. caballus*	Poconé	Mato Grosso	16°15'S 56°37'W	April 2002	CNC 647
A. sculptum	1	6	*E. caballus*	Poconé	Mato Grosso	16°15'S 56°37'W	October 2012	CNC 2315
A. sculptum	13	22	*E. caballus*	Poconé	Mato Grosso	16°15'S 56°37'W	July 2011	CNC 2648
A. sculptum	8	12	*H. sapiens*	Poconé	Mato Grosso	16°15'S 56°37'W	April 2002	CNC 649
A. sculptum		1	*P. onca*	Poconé	Mato Grosso	16°15'S 56°37'W	August 1996	CNC 144
A. sculptum	1	2	*P. onca*	Poconé	Mato Grosso	16°15'S 56°37'W	November 2008	CNC 1358
A. sculptum		1	*P. onca*	Poconé	Mato Grosso	16°15'S 56°37'W	June 2011	CNC 1921
A. sculptum	2	1	*P. onca*	Poconé	Mato Grosso	16°15'S 56°37'W	June 2013	CNC 2495
A. sculptum		1	*P. onca*	Anaurilândia	Mato Grosso do Sul	22°11'S 52°43'W	March 2001	CNC 469
A. sculptum	1	3	*P. onca*	Anaurilândia	Mato Grosso do Sul	22°11'S 52°43'W	February 2002	CNC 833
A. sculptum	3	1	*P. tajacu*	Anaurilândia	Mato Grosso do Sul	22°11'S 52°43'W	August 2000	CNC 326
A. sculptum	11	28	*T. terrestris*	Aquidauana	Mato Grosso do Sul	20°28'S 55°47'W	September 2008	CNC 1146
A. sculptum	8	21	*T. terrestris*	Aquidauana	Mato Grosso do Sul	20°28'S 55°47'W	no data	CNC 1424
A. sculptum	14	56	*T. terrestris*	Aquidauana	Mato Grosso do Sul	20°28'S 55°47'W	July 2009	CNC 1456
A. sculptum		17	*T. terrestris*	Aquidauana	Mato Grosso do Sul	20°28'S 55°47'W	August 2010	CNC 1651
A. sculptum	4	18	*T. terrestris*	Aquidauana	Mato Grosso do Sul	20°28'S 55°47'W	May 2011	CNC 1915
A. sculptum	8	14	*T. terrestris*	Aquidauana	Mato Grosso do Sul	20°28'S 55°47'W	August 2011	CNC 1937
A. sculptum	6	24	*T. terrestris*	Aquidauana	Mato Grosso do Sul	20°28'S 55°47'W	December 2011	CNC 2093
A. sculptum		33	*T. terrestris*	Aquidauana	Mato Grosso do Sul	20°28'S 55°47'W	May 2012	CNC 2181
A. sculptum	23	89	*T. terrestris*	Aquidauana	Mato Grosso do Sul	20°28'S 55°47'W	November 2012	CNC 2304

A. sculptum		36	*T. terrestris*	Aquidauana	Mato Grosso do Sul	20°28’S 55°47’W	May 2013	CNC 2478
A. sculptum	6	30	*T. terrestris*	Aquidauana	Mato Grosso do Sul	20°28’S 55°47’W	July 2013	CNC 2512
A. sculptum		13	*T. terrestris*	Aquidauana	Mato Grosso do Sul	20°28’S 55°47’W	December 2013	CNC 2583
A. sculptum	1	1	*B. dichotomus*	Bataguassu	Mato Grosso do Sul	21°42’S 52°25’W	February 2001	CNC 420
A. sculptum		1	*B. dichotomus*	Bataguassu	Mato Grosso do Sul	21°42’S 52°25’W	February 2001	CNC 434
A. sculptum		1	*B. dichotomus*	Bataguassu	Mato Grosso do Sul	21°42’S 52°25’W	no data	CNC 435
A. sculptum	2	2	*M. tridactyla*	Bataguassu	Mato Grosso do Sul	21°42’S 52°25’W	August 2000	CNC 341
A. sculptum	2	3	*M. tridactyla*	Brasilândia	Mato Grosso do Sul	21°15’S 52°2’W	August 2000	CNC 339
A. sculptum		2	*C. brachyurus*	Bonito	Mato Grosso do Sul	21°7’S 56°28’W	March 2002	CNC 623
A. sculptum		1	*H. sapiens*	Bonito	Mato Grosso do Sul	21°7’S 56°28’W	April 2005	CNC 897
A. sculptum	1	1	*T. terrestris*	Bonito	Mato Grosso do Sul	21°7’S 56°28’W	March 2002	CNC 625
A. sculptum		8	*T. terrestris*	Bonito	Mato Grosso do Sul	21°7’S 56°28’W	March 2002	CNC 658
A. sculptum	3	3	*E. caballus*	Campo Grande	Mato Grosso do Sul	20°26’S 54°38’W	December 2012	CNC 2322
A. sculptum		3	*E. caballus*	Corumbá	Mato Grosso do Sul	19°0’S 57°39’W	January 2009	CNC 1364
A. sculptum	17	8	*E. caballus*	Corumbá	Mato Grosso do Sul	19°0’S 57°39’W	April 2009	CNC 1415
A. sculptum	2	2	*E. caballus*	Corumbá	Mato Grosso do Sul	19°0’S 57°39’W	May 2009	CNC 1425
A. sculptum	5	10	*H. sapiens*	Corumbá	Mato Grosso do Sul	19°0’S 57°39’W	January 2009	CNC 1365
A. sculptum	1	3	*H. sapiens*	Corumbá	Mato Grosso do Sul	19°0’S 57°39’W	May 2008	CNC 1404
A. sculptum		2	*H. sapiens*	Corumbá	Mato Grosso do Sul	19°0’S 57°39’W	June 2009	CNC 1430
A. sculptum		1	*H. hydrochaeris*	Corumbá	Mato Grosso do Sul	19°0’S 57°39’W	September 1997	CNC 103
A. sculptum	6	3	*H. hydrochaeris*	Corumbá	Mato Grosso do Sul	19°0’S 57°39’W	August 2008	CNC 1360
A. sculptum	11	38	*P. onca*	Corumbá	Mato Grosso do Sul	19°0’S 57°39’W	June 2011	CNC 2073
A. sculptum	2	2	*P. onca*	Corumbá	Mato Grosso do Sul	19°0’S 57°39’W	October 2012	CNC 2302
A. sculptum	8	5	*Sus scrofa*	Corumbá	Mato Grosso do Sul	19°0’S 57°39’W	August 2010	CNC 1643
A. sculptum	27	11	*Sus scrofa*	Corumbá	Mato Grosso do Sul	19°0’S 57°39’W	July 2011	CNC 1949
A. sculptum	24	10	*Sus scrofa*	Corumbá	Mato Grosso do Sul	19°0’S 57°39’W	August 2010	CNC 2649
A. sculptum	4	1	*T. pecari*	Corumbá	Mato Grosso do Sul	19°0’S 57°39’W	September 1997	CNC 94
A. sculptum	3	3	*T. pecari*	Corumbá	Mato Grosso do Sul	19°0’S 57°39’W	September 1997	CNC 96
A. sculptum	1	2	*T. pecari*	Corumbá	Mato Grosso do Sul	19°0’S 57°39’W	September 1997	CNC 97
A. sculptum	8	2	*T. pecari*	Corumbá	Mato Grosso do Sul	19°0’S 57°39’W	September 1997	CNC 98
A. sculptum	9	7	*T. pecari*	Corumbá	Mato Grosso do Sul	19°0’S 57°39’W	July 2011	CNC 1947
A. sculptum	16	21	free living	Corumbá	Mato Grosso do Sul	19°0’S 57°39’W	July 2011	CNC 1950
A. sculptum		1	*C. unicinctus*	Miranda	Mato Grosso do Sul	20°14’S 56°22’W	December 2012	CNC 2377
A. sculptum		1	*E. sexcinctus*	Miranda	Mato Grosso do Sul	20°14’S 56°22’W	July 2012	CNC 2378
A. sculptum		2	*E. sexcinctus*	Miranda	Mato Grosso do Sul	20°14’S 56°22’W	June 2013	CNC 2498
A. sculptum		1	*E. sexcinctus*	Miranda	Mato Grosso do Sul	20°14’S 56°22’W	December 2013	CNC 2602
A. sculptum		1	*H. sapiens*	Miranda	Mato Grosso do Sul	20°14’S 56°22’W	June 2012	CNC 2215
A. sculptum	2	5	*M. tridactyla*	Miranda	Mato Grosso do Sul	20°14’S 56°22’W	July 2013	CNC 2511
A. sculptum	11	7	*M. tridactyla*	Miranda	Mato Grosso do Sul	20°14’S 56°22’W	December 2013	CNC 2604

A. sculptum	1	4	*P. onca*	Miranda	Mato Grosso do Sul	20°14′S 56°22′W	October 2003	CNC 1675
A. sculptum	1	2	*P. onca*	Miranda	Mato Grosso do Sul	20°14′S 56°22′W	December 2014	CNC 1678
A. sculptum	15	45	*P. onca*	Miranda	Mato Grosso do Sul	20°14′S 56°22′W	December 2015	CNC 1693
A. sculptum	10	26	*P. onca*	Miranda	Mato Grosso do Sul	20°14′S 56°22′W	January 2007	CNC 1702
A. sculptum	1	1	*P. onca*	Miranda	Mato Grosso do Sul	20°14′S 56°22′W	October 2011	CNC 2173
A. sculptum	4	6	*P. onca*	Miranda	Mato Grosso do Sul	20°14′S 56°22′W	April 2012	CNC 2216
A. sculptum	16	20	*P. onca*	Miranda	Mato Grosso do Sul	20°14′S 56°22′W	April 2013	CNC 2452
A. sculptum		1	*P. maximus*	Miranda	Mato Grosso do Sul	20°14′S 56°22′W	July 2010	CNC 1636
A. sculptum	24	19	*P. maximus*	Miranda	Mato Grosso do Sul	20°14′S 56°22′W	July 2012	CNC 2379
A. sculptum	36	36	*P. maximus*	Miranda	Mato Grosso do Sul	20°14′S 56°22′W	April 2013	CNC 2465
A. sculptum	42	32	*P. maximus*	Miranda	Mato Grosso do Sul	20°14′S 56°22′W	June 2013	CNC 2499
A. sculptum	2	1	*P. maximus*	Miranda	Mato Grosso do Sul	20°14′S 56°22′W	August 2013	CNC 2516
A. sculptum	5	7	*P. maximus*	Miranda	Mato Grosso do Sul	20°14′S 56°22′W	November 2013	CNC 2546
A. sculptum	2	6	*P. maximus*	Miranda	Mato Grosso do Sul	20°14′S 56°22′W	December 2013	CNC 2603
A. sculptum	1	2	free living	Miranda	Mato Grosso do Sul	20°14′S 56°22′W	July 2010	CNC 1645
A. sculptum	9	2	*M. tridactyla*	Nova Alvorada do Sul	Mato Grosso do Sul	21°27′S 54°23′W	April 2005	CNC 895
A. sculptum	4	4	*T. terrestris*	Nova Alvorada do Sul	Mato Grosso do Sul	21°27′S 54°23′W	August 2013	CNC 2513
A. sculptum	6	3	*M. tridactyla*	Santa Rita do Pardo	Mato Grosso do Sul	21°18′S 52°49′W	July 2000	CNC 337
A. sculptum	3	1	*M. tridactyla*	Santa Rita do Pardo	Mato Grosso do Sul	21°18′S 52°49′W	August 2000	CNC 347
A. sculptum		1	*T. tetradactyla*	Santa Rita do Pardo	Mato Grosso do Sul	21°18′S 52°49′W	August 2000	CNC 352
A. sculptum	17	37	free living	Terenos	Mato Grosso do Sul	20°26′S 54°51′W	November 2012	CNC 2372
A. sculptum	1	1	*H. sapiens*	Três Lagoas	Mato Grosso do Sul	20°45′S 51°40′W	March 1999	CNC 215
A. sculptum	3	2	free living	Colatina	Espírito Santo	19°32′S 40°37′W	October 2007	CNC 1063
A. sculptum	5	7	free living	Nova Venécia	Espírito Santo	18°42′S 40°24′W	May 2007	CNC 1045
A. sculptum	4		*E. caballus*	Pinheiros	Espírito Santo	18°24′S 40°13′W	July 2011	CNC 2645
A. sculptum		7	*E. caballus*	Araguari	Minas Gerais	18°38′S 48°11′W	May 2011	CNC 1909
A. sculptum	5	25	*E. caballus*	Araguari	Minas Gerais	18°38′S 48°11′W	May 2011	CNC 2650
A. sculptum	6	5	*C. brachyurus*	Chapada Gaúcha	Minas Gerais	15°18′S 45°37′W	September 2009	CNC 1455
A. sculptum	1	3	*H. sapiens*	Chapada Gaúcha	Minas Gerais	15°18′S 45°37′W	December 2007	CNC 1414
A. sculptum		1	*H. sapiens*	Chapada Gaúcha	Minas Gerais	15°18′S 45°37′W	May 2012	CNC 2175
A. sculptum		2	*P. onca*	Chapada Gaúcha	Minas Gerais	15°18′S 45°37′W	August 2011	CNC 1956
A. sculptum	2	1	free living	Chapada Gaúcha	Minas Gerais	15°18′S 45°37′W	January 2009	CNC 1441
A. sculptum	1	3	free living	Chapada Gaúcha	Minas Gerais	15°18′S 45°37′W	May 2012	CNC 2176
A. sculptum	5	3	free living	Chapada Gaúcha	Minas Gerais	15°18′S 45°37′W	May 2012	CNC 2177
A. sculptum	6	21	free living	Chapada Gaúcha	Minas Gerais	15°18′S 45°37′W	May 2012	CNC 2179
A. sculptum		1	free living	Doresópolis	Minas Gerais	20°17′S 45°54′W	August 2008	CNC 1341
A. sculptum	4	1	*H. hydrochaeris*	Guarda-Mor	Minas Gerais	17°46′S 47°5′W	August 2013	CNC 2519
A. sculptum		1	*C. familiaris*	Itabira	Minas Gerais	19°37′S 43°13′W	December 2010	CNC 1780
A. sculptum	30	20	*E. caballus*	Itabira	Minas Gerais	19°37′S 43°13′W	January 2011	CNC 2651

A. sculptum	2	4	*H. sapiens*	Marliéria	Minas Gerais	19°42'S 42°43'W	September 2012	CNC 2266
A. sculptum	1	2	*M. tridactyla*	Monte Carmelo	Minas Gerais	18°43'S 47°29'W	November 2013	CNC 2543
A. sculptum		1	*D. azarae*	Morada Nova de Minas	Minas Gerais	18°36'S 45°21'W	May 2009	CNC 2083
A. sculptum	21	11	free living	Morada Nova de Minas	Minas Gerais	18°36'S 45°21'W	May 2009	CNC 1885
A. sculptum	2	1	free living	Pedra Bonita	Minas Gerais	20°31'S 42°19'W	April 2013	CNC 2467
A. sculptum	1	2	*M. tridactyla*	Pratinha	Minas Gerais	19°45'S 46°22'W	May 2008	CNC 1114
A. sculptum	147	110	*H. hydrochaeris*	Santana do Riacho	Minas Gerais	19°10'S 43°42'W	September 2010	CNC 1734
A. sculptum	3	1	*B. taurus*	São Roque de Minas	Minas Gerais	20°14'S 46°21'W	April 2007	CNC 1018
A. sculptum	3	3	*C. brachyurus*	São Roque de Minas	Minas Gerais	20°14'S 46°21'W	September 2007	CNC 1362
A. sculptum	7	16	*S. scrofa*	São Roque de Minas	Minas Gerais	20°14'S 46°21'W	March 2008	CNC 1096
A. sculptum	25	36	free living	São Roque de Minas	Minas Gerais	20°14'S 46°21'W	April 2007	CNC 1016
A. sculptum	3	10	free living	São Roque de Minas	Minas Gerais	20°14'S 46°21'W	August 2007	CNC 1046
A. sculptum	4	3	free living	São Roque de Minas	Minas Gerais	20°14'S 46°21'W	March 2008	CNC 1099
A. sculptum	2	1	free living	São Roque de Minas	Minas Gerais	20°14'S 46°21'W	May 2008	CNC 1115
A. sculptum	8	8	free living	São Roque de Minas	Minas Gerais	20°14'S 46°21'W	June 2007	CNC 1149
A. sculptum	14	21	free living	São Roque de Minas	Minas Gerais	20°14'S 46°21'W	February 2009	CNC 1417
A. sculptum	1	4	free living	São Roque de Minas	Minas Gerais	20°14'S 46°21'W	May 2009	CNC 1495
A. sculptum	1	4	free living	São Roque de Minas	Minas Gerais	20°14'S 46°21'W	September 2011	CNC 2048
A. sculptum	3	2	*E. caballus*	Tiradentes	Minas Gerais	21°6'S 44°10'W	January 2008	CNC 1083
A. sculptum	6	5	*H. sapiens*	Três Marias	Minas Gerais	18°12'S 45°14'W	September 2013	CNC 2529
A. sculptum	23	39	free living	Vargem Bonita	Minas Gerais	20°19'S 46°21'W	March 2008	CNC 1100
A. sculptum	10	9	free living	Vargem Bonita	Minas Gerais	20°19'S 46°21'W	May 2008	CNC 1116
A. sculptum	28	26	free living	Vargem Bonita	Minas Gerais	20°19'S 46°21'W	August 2008	CNC 1150
A. sculptum	63	123	free living	Vargem Bonita	Minas Gerais	20°19'S 46°21'W	February 2009	CNC 1418
A. sculptum	32	26	free living	Vargem Bonita	Minas Gerais	20°19'S 46°21'W	December 2011	CNC 2652
A. sculptum	6	6	*E. caballus*	Seropédica	Rio de Janeiro	22°44'S 43°42'W	May 2011	CNC 2653
A. sculptum	2	4	*T. tetradactyla*	Teresópolis	Rio de Janeiro	22°24'S 42°57'W	September 2009	CNC 1793
A. sculptum	14	22	free living	Americana	São Paulo	22°44'S 47°19'W	September 2011	CNC 1957
A. sculptum	21	20	free living	Americana	São Paulo	22°44'S 47°19'W	October 2011	CNC 2070
A. sculptum	32	39	free living	Americana	São Paulo	22°44'S 47°19'W	December 2011	CNC 2088
A. sculptum	29	64	free living	Americana	São Paulo	22°44'S 47°19'W	May 2005	CNC 2654
A. sculptum	2	8	*H. hydrochaeris*	Andradina	São Paulo	20°53'S 51°22'W	September 2005	CNC 962
A. sculptum		1	*M. tridactyla*	Angatuba	São Paulo	23°29'S 48°24'W	October 2010	CNC 1739
A. sculptum		1	*C. familiaris*	Araçariguama	São Paulo	23°26'S 47°3'W	November 1997	CNC 108
A. sculptum	1	1	*C. familiaris*	Araçariguama	São Paulo	23°26'S 47°3'W	January 1998	CNC 116
A. sculptum		1	*H. hydrochaeris*	Araçariguama	São Paulo	23°26'S 47°3'W	July 2013	CNC 2514
A. sculptum		1	free living	Araçariguama	São Paulo	23°26'S 47°3'W	March 1998	CNC 129
A. sculptum		1	*S. villosus*	Biritiba Mirim	São Paulo	23°34'S 46°2'W	March 2005	CNC 1119
A. sculptum		1	*H. hydrochaeris*	Bragança Paulista	São Paulo	22°57'S 46°32'W	October 2003	CNC 809

A. sculptum	2	5	free living	Campinas	São Paulo	22°54'S 47°3'W	March 2009	CNC 1406
A. sculptum		1	*E. caballus*	Franca	São Paulo	20°32'S 47°24'W	March 2001	CNC 520
A. sculptum	1	3	*H. hydrochaeris*	Guarulhos	São Paulo	23°27'S 46°31'W	March 2005	CNC 961
A. sculptum	18	6	*E. caballus*	Indaiatuba	São Paulo	23°5'S 47°13'W	November 2003	CNC 808
A. sculptum		1	*M. tridactyla*	Itapetininga	São Paulo	23°35'S 48°3'W	January 2008	CNC 1086
A. sculptum	12	23	*H. hydrochaeris*	Itu	São Paulo	23°15'S 47°17'W	September 2008	CNC 1537
A. sculptum		1	*H. hydrochaeris*	Itu	São Paulo	23°15'S 47°17'W	November 2010	CNC 1749
A. sculptum	3	6	*M. tridactyla*	Itu	São Paulo	23°15'S 47°17'W	July 2008	CNC 1132
A. sculptum	1	16	*M. tridactyla*	Itu	São Paulo	23°15'S 47°17'W	November 2009	CNC 1503
A. sculptum	40	46	free living	Itu	São Paulo	23°15'S 47°17'W	January 2006	CNC 957
A. sculptum		1	*H. sapiens*	Jarinu	São Paulo	23°6'S 46° 43'W	February 2009	CNC 1368
A. sculptum		1	*Cariama cristata*	Jundiaí	São Paulo	23°11'S 46°53'W	November 2001	CNC 687
A. sculptum		1	*H. hydrochaeris*	Jundiaí	São Paulo	23°11'S 46°53'W	April 2000	CNC 692
A. sculptum		6	*E. caballus*	Lorena	São Paulo	22°43'S 45°7'W	January 2013	CNC 2343
A. sculptum		1	*P. tajacu*	Luís Antônio	São Paulo	21°33'S 47°42'W	July 2002	CNC 738
A. sculptum	270	237	free living	Mogi das Cruzes	São Paulo	23°31'S 46°11'W	October 2005	CNC 964
A. sculptum	83	65	free living	Panorama	São Paulo	21°21'S 51°51'W	November 2002	CNC 747
A. sculptum		1	*B. dichotomus*	Pauliceia	São Paulo	21°19'S 51°49'W	April 2001	CNC 505
A. sculptum	1	8	*E. caballus*	Pauliceia	São Paulo	21°19'S 51°49'W	April 2001	CNC 454
A. sculptum	1	1	*E. caballus*	Pauliceia	São Paulo	21°19'S 51°49'W	April 2001	CNC 513
A. sculptum	4	14	*E. caballus*	Pauliceia	São Paulo	21°19'S 51°49'W	January 2002	CNC 592
A. sculptum		1	*E. caballus*	Pauliceia	São Paulo	21°19'S 51°49'W	April 2002	CNC 641
A. sculptum	19	19	free living	Pauliceia	São Paulo	21°19'S 51°49'W	July 2001	CNC 532
A. sculptum	32	41	free living	Pauliceia	São Paulo	21°19'S 51°49'W	January 2002	CNC 588
A. sculptum		1	free living	Pauliceia	São Paulo	21°19'S 51°49'W	January 2002	CNC 589
A. sculptum	12	6	free living	Pauliceia	São Paulo	21°19'S 51°49'W	January 2002	CNC 590
A. sculptum	14	37	free living	Pauliceia	São Paulo	21°19'S 51°49'W	January 2002	CNC 591
A. sculptum	16	8	free living	Pauliceia	São Paulo	21°19'S 51°49'W	April 2002	CNC 636
A. sculptum	3	7	free living	Pauliceia	São Paulo	21°19'S 51°49'W	April 2002	CNC 638
A. sculptum	16	26	free living	Pauliceia	São Paulo	21°19'S 51°49'W	April 2002	CNC 639
A. sculptum	6	9	free living	Pauliceia	São Paulo	21°19'S 51°49'W	April 2002	CNC 640
A. sculptum	2	4	free living	Pauliceia	São Paulo	21°19'S 51°49'W	April 2002	CNC 642
A. sculptum	5	5	free living	Pedreira	São Paulo	22°44'S 46°54'W	February 2011	CNC 2655
A. sculptum	3	1	*E. caballus*	Peruíbe	São Paulo	24°19'S 46°59'W	February 2011	CNC 1813
A. sculptum		1	free living	Peruíbe	São Paulo	24°19'S 46°59'W	November 2010	CNC 1855
A. sculptum	5	6	*H. hydrochaeris*	Piracicaba	São Paulo	22°43'S 47°38'W	March 2001	CNC 436
A. sculptum	27	18	*H. hydrochaeris*	Piracicaba	São Paulo	22°43'S 47°38'W	October 2001	CNC 566
A. sculptum	1	4	*E. caballus*	Pirassununga	São Paulo	21°59'S 47°25'W	May 1997	CNC 16
A. sculptum	2	8	*E. caballus*	Pirassununga	São Paulo	21°59'S 47°25'W	December 2008	CNC 1809

A. sculptum		1	free living	Pirassununga	São Paulo	21°59'S 47°25'W	October 2000	CNC 363
A. sculptum	3	9	*E. caballus*	Presidente Prudente	São Paulo	22°7'S 51°23'W	January 2006	CNC 1005
A. sculptum	1	2	*E. caballus*	Ribeirão Grande	São Paulo	24°5'S 48°21'W	December 2001	CNC 605
A. sculptum	3	5	free living	Ribeirão Grande	São Paulo	24°5'S 48°21'W	December 2005	CNC 989
A. sculptum	30	9	*H. hydrochaeris*	Ribeirão Preto	São Paulo	21°10'S 47°48'W	March 2005	CNC 960
A. sculptum	5	2	*P. tajacu*	Santa Rita do Passa Quatro	São Paulo	21°42'S 47°28'W	March 2003	CNC 762
A. sculptum		1	*D. novemcinctus*	São Paulo	São Paulo	23°32'S 46°38'W	January 2013	CNC 2424
A. sculptum		1	*H. hydrochaeris*	São Paulo	São Paulo	23°32'S 46°38'W	July 2005	CNC 2435
A. sculptum	1	3	*H. hydrochaeris*	São Paulo	São Paulo	23°32'S 46°38'W	June 2013	CNC 2487
A. sculptum		1	*Nasua nasua*	São Paulo	São Paulo	23°32'S 46°38'W	June 2013	CNC 2484
A. sculptum		1	*T. terrestris*	São Paulo	São Paulo	23°32'S 46°38'W	February 2010	CNC 2423
A. sculptum	118	33	*P. tajacu*	Sete Barras	São Paulo	24°23'S 47°55'W	February 2013	CNC 2427
A. sculptum	1	1	*C. brachyurus*	Sorocaba	São Paulo	23°30'S 47°27'W	August 1997	CNC 92
A. sculptum		1	*H. hydrochaeris*	Sorocaba	São Paulo	23°30'S 47°27'W	June 2007	CNC 1049
A. sculptum	2	2	*H. hydrochaeris*	Sorocaba	São Paulo	23°30'S 47°27'W	December 2009	CNC 1502
A. sculptum	11	2	*H. hydrochaeris*	Sorocaba	São Paulo	23°30'S 47°27'W	December 2013	CNC 2588
A. sculptum		1	*M. tridactyla*	Sorocaba	São Paulo	23°30'S 47°27'W	December 2012	CNC 2300
A. sculptum		1	*L. Pardalis*	Teodoro Sampaio	São Paulo	22°31'S 52°10'W	November 1999	CNC 1439
A. sculptum		8	*P. onca*	Teodoro Sampaio	São Paulo	22°31'S 52°10'W	July 2003	CNC 790
A. sculptum	8	9	*P. tajacu*	Teodoro Sampaio	São Paulo	22°31'S 52°10'W	July 2002	CNC 753
A. sculptum	1	1	*P. tajacu*	Teodoro Sampaio	São Paulo	22°31'S 52°10'W	June 2002	CNC 757
A. sculptum	9	3	*P. tajacu*	Teodoro Sampaio	São Paulo	22°31'S 52°10'W	June 2002	CNC 759
A. sculptum	2	1	*P. tajacu*	Teodoro Sampaio	São Paulo	22°31'S 52°10'W	December 2003	CNC 805
A. sculptum	17	13	*P. tajacu*	Teodoro Sampaio	São Paulo	22°31'S 52°10'W	January 2004	CNC 856
A. sculptum	6	2	*T. terrestris*	Teodoro Sampaio	São Paulo	22°31'S 52°10'W	January 2007	CNC 1001
A. sculptum	5	7	*T. terrestris*	Teodoro Sampaio	São Paulo	22°31'S 52°10'W	January 2007	CNC 1002
A. sculptum	1	5	*T. terrestris*	Teodoro Sampaio	São Paulo	22°31'S 52°10'W	January 2007	CNC 1003
A. sculptum	5	9	*T. terrestris*	Teodoro Sampaio	São Paulo	22°31'S 52°10'W	January 2007	CNC 1004
A. sculptum	2	3	*T. terrestris*	Teodoro Sampaio	São Paulo	22°31'S 52°10'W	June 2007	CNC 1032
A. sculptum	8	9	*T. pecari*	Teodoro Sampaio	São Paulo	22°31'S 52°10'W	April 2002	CNC 653
A. sculptum	1	5	*T. pecari*	Teodoro Sampaio	São Paulo	22°31'S 52°10'W	March 2004	CNC 839
A. sculptum	49	27	*T. pecari*	Teodoro Sampaio	São Paulo	22°31'S 52°10'W	March 2005	CNC 904
A. sculptum	12	16	*T. pecari*	Teodoro Sampaio	São Paulo	22°31'S 52°10'W	October 2005	CNC 930
A. sculptum	5	1	*T. pecari*	Teodoro Sampaio	São Paulo	22°31'S 52°10'W	July 2001	CNC 1029
A. sculptum	1	3	*T. pecari*	Teodoro Sampaio	São Paulo	22°31'S 52°10'W	January 2001	CNC 1031
A. sculptum		1	free living	Teodoro Sampaio	São Paulo	22°31'S 52°10'W	September 2000	CNC 360
A. sculptum	3	1	free living	Teodoro Sampaio	São Paulo	22°31'S 52°10'W	March 2005	CNC 905
A. sculptum	24	19	free living	Teodoro Sampaio	São Paulo	22°31'S 52°10'W	July 2005	CNC 931
A. sculptum	12	10	free living	Teodoro Sampaio	São Paulo	22°31'S 52°10'W	February 2006	CNC 988

A. sculptum	22	8	free living	Teodoro Sampaio	São Paulo	22°31'S 52°10'W	November 2006	CNC 1213
A. sculptum	5	4	free living	Teodoro Sampaio	São Paulo	22°31'S 52°10'W	October 2005	CNC 1236
A. sculptum	2	1	free living	Teodoro Sampaio	São Paulo	22°31'S 52°10'W	April 2006	CNC 1239
A. sculptum	33	26	free living	Teodoro Sampaio	São Paulo	22°31'S 52°10'W	November 2006	CNC 1250
A. sculptum	1	1	free living	Ubatuba	São Paulo	23°26'S 45°4'W	January 2005	CNC 880
A. sculptum		4	*E. caballus*	Alvorada do Sul	Paraná	22°46'S 51°13'W	May 2012	CNC3157
A. sculptum		21	*E. caballus*	Londrina	Paraná	23°18'S 51°9'W	June 2004	CNC 2656
A. sculptum		1	*H. sapiens*	Londrina	Paraná	23°18'S 51°9'W	December 2013	CNC 2600
A. sculptum	1	1	*T. terrestris*	Foz do Iguaçu	Paraná	25°32'S 54°35'W	March 1998	CNC 126
A. sculptum	1	1	*E. caballus*	Rosário do Ivaí	Paraná	24°16'S 51°16'W	January 2012	CNC 2657
*A. sculptum**		9	*C. familiaris*	Cristalândia	Tocantins	10°36'S 49°11'W	August 2011	CNC 2619
*A. sculptum**	3	3	*E. caballus*	Balsas	Maranhão	7°31'S 46°2'W	July 2011	CNC 2631
*A. sculptum**	11	5	*C. thous*	Barão de Melgaço	Mato Grosso	16°11'S 55°58'W	September 2002	CNC 739
*A. sculptum**	2	2	*C. thous*	Barão de Melgaço	Mato Grosso	16°11'S 55°58'W	July 2003	CNC 789
*A. sculptum**	24	20	*C. thous*	Barão de Melgaço	Mato Grosso	16°11'S 55°58'W	August 2003	CNC 791
*A. sculptum**		3	*C. thous*	Barão de Melgaço	Mato Grosso	16°11'S 55°58'W	October 2002	CNC 793
*A. sculptum**	3	1	*C. thous*	Barão de Melgaço	Mato Grosso	16°11'S 55°58'W	January 2004	CNC 828
*A. sculptum**	4	2	*C. thous*	Barão de Melgaço	Mato Grosso	16°11'S 55°58'W	March 2004	CNC 841
*A. sculptum**	25	49	*C. thous*	Barão de Melgaço	Mato Grosso	16°11'S 55°58'W	September 2004	CNC 870
*A. sculptum**	26	49	*C. brachyurus*	Barão de Melgaço	Mato Grosso	16°11'S 55°58'W	August 2002	CNC 731
*A. sculptum**	4	9	*C. brachyurus*	Barão de Melgaço	Mato Grosso	16°11'S 55°58'W	September 2002	CNC 741
*A. sculptum**	8	22	*C. brachyurus*	Barão de Melgaço	Mato Grosso	16°11'S 55°58'W	October 2002	CNC 794
*A. sculptum**	15	26	*C. brachyurus*	Barão de Melgaço	Mato Grosso	16°11'S 55°58'W	December 2003	CNC 804
*A. sculptum**	7	12	*C. brachyurus*	Barão de Melgaço	Mato Grosso	16°11'S 55°58'W	August 2006	CNC 993
*A. sculptum**	44	76	*L. pardalis*	Barão de Melgaço	Mato Grosso	16°11'S 55°58'W	July 2004	CNC 869
*A. sculptum**	3	10	*P. cancrivorus*	Barão de Melgaço	Mato Grosso	16°11'S 55°58'W	July 2002	CNC 716
*A. sculptum**	26	11	*P. cancrivorus*	Barão de Melgaço	Mato Grosso	16°11'S 55°58'W	August 2002	CNC 730
*A. sculptum**	3	6	*P. cancrivorus*	Barão de Melgaço	Mato Grosso	16°11'S 55°58'W	September 2002	CNC 740
*A. sculptum**	2	4	*P. cancrivorus*	Barão de Melgaço	Mato Grosso	16°11'S 55°58'W	October 2003	CNC 803
*A. sculptum**	1	1	*S. venaticus*	Barão de Melgaço	Mato Grosso	16°11'S 55°58'W	July 2004	CNC 853
*A. sculptum**		2	*S. venaticus*	Barão de Melgaço	Mato Grosso	16°11'S 55°58'W	August 2006	CNC 994
*A. sculptum**	6	2	*C. thous*	Chapada dos Guimarães	Mato Grosso	15°27'S 55°45'W	August 2001	CNC 551
*A. sculptum**	4	2	*C. thous*	Chapada dos Guimarães	Mato Grosso	15°27'S 55°45'W	June 2001	CNC 552
*A. sculptum**	8	9	*C. thous*	Chapada dos Guimarães	Mato Grosso	15°27'S 55°45'W	June 2001	CNC 553
*A. sculptum**	4	7	*C. thous*	Chapada dos Guimarães	Mato Grosso	15°27'S 55°45'W	August 2001	CNC 674
*A. sculptum**		1	*C. thous*	Água Clara	Mato Grosso do Sul	20°26'S 52°52'W	August 2011	CNC 1946
*A. sculptum**		1	*P. cancrivorus*	Anastácio	Mato Grosso do Sul	20°29'S 55°48'W	August 2011	CNC 1945
*A. sculptum**	2	6	*P. concolor*	Anaurilândia	Mato Grosso do Sul	22°11'S 52°43'W	June 2001	CNC 530
*A. sculptum**	18	13	*M. tridactyla*	Aquidauana	Mato Grosso do Sul	20°28'S 55°47'W	November 2013	CNC 2542

*A. sculptum**	2	22	*T. terrestris*	Aquidauana	Mato Grosso do Sul	20°28’S 55°47’W	May 2010	CNC 1588
*A. sculptum**	30	53	*T. terrestris*	Aquidauana	Mato Grosso do Sul	20°28’S 55°47’W	September 2011	CNC 1960
*A. sculptum**	47	104	*T. terrestris*	Aquidauana	Mato Grosso do Sul	20°28’S 55°47’W	November 2011	CNC 2080
*A. sculptum**	47	71	*T. terrestris*	Aquidauana	Mato Grosso do Sul	20°28’S 55°47’W	July 2012	CNC 2237
*A. sculptum**	32	69	*T. terrestris*	Aquidauana	Mato Grosso do Sul	20°28’S 55°47’W	September 2012	CNC 2268
*A. sculptum**	23	20	*T. terrestris*	Aquidauana	Mato Grosso do Sul	20°28’S 55°47’W	October 2013	CNC 2538
*A. sculptum**	3	2	*M. gouazoubira*	Brasilândia	Mato Grosso do Sul	21°15’S 52°2’W	August 2000	CNC 327
*A. sculptum**	1	2	*M. tridactyla*	Brasilândia	Mato Grosso do Sul	21°15’S 52°2’W	July 2000	CNC 332
*A. sculptum**	17	28	*P. tajacu*	Corumbá	Mato Grosso do Sul	19°0’S 57°39’W	September 1997	CNC 99
*A. sculptum**	1	1	*P. tajacu*	Corumbá	Mato Grosso do Sul	19°0’S 57°39’W	September 1997	CNC 100
*A. sculptum**	12	6	*M. tridactyla*	Miranda	Mato Grosso do Sul	20°14’S 56°22’W	July 2011	CNC 1948
*A. sculptum**	6	2	*T. terrestris*	Miranda	Mato Grosso do Sul	20°14’S 56°22’W	June 2008	CNC 1123
*A. sculptum**	4	7	*T. terrestris*	Miranda	Mato Grosso do Sul	20°14’S 56°22’W	July 2010	CNC 1637
*A. sculptum**		1	*H. sapiens*	Colatina	Espírito Santo	19°32’S 40°37’W	September 2007	CNC 1069
*A. sculptum**	47	53	free living	Colatina	Espírito Santo	19°32’S 40°37’W	October 2007	CNC 1082
*A. sculptum**	2	7	*E. caballus*	Guarapari	Espírito Santo	20°39’S 40°30’W	July 2001	CNC 529
*A. sculptum**	3	4	*C. familiaris*	Nova Venécia	Espírito Santo	18°42’S 40°24’W	September 2007	CNC 1055
*A. sculptum**	12	17	*E. caballus*	Nova Venécia	Espírito Santo	18°42’S 40°24’W	September 2007	CNC 1056
*A. sculptum**	2	1	*C. familiaris*	Santana do Riacho	Minas Gerais	19°10’S 43°42’W	September 2012	CNC 2253
*A. sculptum**	5	11	*E. caballus*	Santana do Riacho	Minas Gerais	19°10’S 43°42’W	September 2012	CNC 2252
*A. sculptum**		4	*C. brachyurus*	São Roque de Minas	Minas Gerais	20°14’S 46°21’W	January 2005	CNC 981
*A. sculptum**	1	1	*C. brachyurus*	São Roque de Minas	Minas Gerais	20°14’S 46°21’W	January 2006	CNC 1013
*A. sculptum**	6	12	*C. brachyurus*	São Roque de Minas	Minas Gerais	20°14’S 46°21’W	October 2006	CNC 1022
*A. sculptum**	3	2	*L. vetulus*	São Roque de Minas	Minas Gerais	20°14’S 46°21’W	October 2005	CNC 980
*A. sculptum**	11	44	*H. hydrochaeris*	Três Rios	Rio de Janeiro	22°7’S 43°12’W	September 2008	CNC 1159
*A. sculptum**		1	*C. familiaris*	Araçariguama	São Paulo	23°26’S 47°3’W	March 2009	CNC 1405
*A. sculptum**	1	2	*C. familiaris*	Araçariguama	São Paulo	23°26’S 47°3’W	March 2009	CNC 1434
*A. sculptum**		1	*C. familiaris*	Campo Limpo Paulista	São Paulo	23°12’S 46°47’W	November 1999	CNC 254
*A. sculptum**	2	2	*T. caudatus*	Franca	São Paulo	20°32’S 47°24’W	July 2002	CNC 729
*A. sculptum**		1	*H. sapiens*	Itu	São Paulo	23°15’S 47°17’W	January 2006	CNC 966
*A. sculptum**	1	5	*M. tridactyla*	Itu	São Paulo	23°15’S 47°17’W	April 2009	CNC 1416
*A. sculptum**		1	*T. terrestris*	Itu	São Paulo	23°15’S 47°17’W	May 2001	CNC 493
*A. sculptum**	2	1	*C. familiaris*	Jarinu	São Paulo	23°6’S 46° 43’W	October 2009	CNC 1454
*A. sculptum**		1	"hawk"	Jundiaí	São Paulo	23°11’S 46°53’W	October 1998	CNC 172
*A. sculptum**	4	6	*M. gouazoubira*	Jundiaí	São Paulo	23°11’S 46°53’W	August 1999	CNC 242
*A. sculptum**		2	*M. gouazoubira*	Jundiaí	São Paulo	23°11’S 46°53’W	August 1999	CNC 243
*A. sculptum**	2	2	*M. gouazoubira*	Jundiaí	São Paulo	23°11’S 46°53’W	November 1999	CNC 276
*A. sculptum**		1	*S. brasiliensis*	Jundiaí	São Paulo	23°11’S 46°53’W	October 1999	CNC 250
*A. sculptum**	1	2	*P. tajacu*	Luís Antônio	São Paulo	21°33’S 47°42’W	August 2001	CNC 565

*A. sculptum**	12	5	free living	Panorama	São Paulo	21°21’S 51°51’W	July 2002	CNC 722
*A. sculptum**		3	*E. caballus*	Pauliceia	São Paulo	21°19’S 51°49’W	July 2002	CNC 720
*A. sculptum**	1	3	free living	Pauliceia	São Paulo	21°19’S 51°49’W	April 2002	CNC 668
*A. sculptum**	2	5	free living	Pauliceia	São Paulo	21°19’S 51°49’W	April 2002	CNC 683
*A. sculptum**	15	20	free living	Pauliceia	São Paulo	21°19’S 51°49’W	April 2002	CNC 684
*A. sculptum**	22	30	free living	Pauliceia	São Paulo	21°19’S 51°49’W	July 2002	CNC 736
*A. sculptum**	5	2	*H. sapiens*	Pedreira	São Paulo	22°44’S 46°54’W	December 2000	CNC 425
*A. sculptum**	1	3	*D. albiventris*	Pirassununga	São Paulo	21°59’S 47°25’W	October 2000	CNC 364
*A. sculptum**	1	2	*C. thous*	Presidente Epitácio	São Paulo	21°45’S 52°6’W	May 2001	CNC 488
*A. sculptum**	8	15	*P. tajacu*	Presidente Epitácio	São Paulo	21°45’S 52°6’W	May 2001	CNC 489
*A. sculptum**	3	12	*M. gouazoubira*	Ribeirão Grande	São Paulo	24°5’S 48°21’W	December 2001	CNC 606
*A. sculptum**	13	11	*C. brachyurus*	Ribeirão Preto	São Paulo	21°10’S 47°48’W	August 1999	CNC 297
*A. sculptum**		1	*C. brachyurus*	Ribeirão Preto	São Paulo	21°10’S 47°48’W	September 2002	CNC 799
*A. sculptum**	2	3	*C. familiaris*	São Luiz do Paraitinga	São Paulo	23°13’S 45°18’W	September 2013	CNC 2523
*A. sculptum**	4	1	*H. hydrochaeris*	São Paulo	São Paulo	23°32’S 46°38’W	August 2012	CNC 713
*A. sculptum**	6	3	*H. hydrochaeris*	São Paulo	São Paulo	23°32’S 46°38’W	June 2006	CNC 983
*A. sculptum**	13	5	*H. hydrochaeris*	São Paulo	São Paulo	23°32’S 46°38’W	June 2006	CNC 984
*A. sculptum**	4	1	*H. hydrochaeris*	São Paulo	São Paulo	23°32’S 46°38’W	June 2006	CNC 985
*A. sculptum**	7	2	*H. hydrochaeris*	São Paulo	São Paulo	23°32’S 46°38’W	June 2006	CNC 986
*A. sculptum**		2	*C. cristata*	Sorocaba	São Paulo	23°30’S 47°27’W	September 2007	CNC 1053
*A. sculptum**	6	34	*M. gouazoubira*	Sorocaba	São Paulo	23°30’S 47°27’W	September 2007	CNC 1054
*A. sculptum**	4	3	*M. gouazoubira*	Sorocaba	São Paulo	23°30’S 47°27’W	September 2007	CNC 1077
*A. sculptum**	1	2	*D. azarae*	Teodoro Sampaio	São Paulo	22°31’S 52°10’W	May 2002	CNC 701
*A. sculptum**		1	*D. azarae*	Teodoro Sampaio	São Paulo	22°31’S 52°10’W	January 2009	CNC 1366
*A. sculptum**	3	3	*T. terrestris*	Teodoro Sampaio	São Paulo	22°31’S 52°10’W	July 2006	CNC 972
*A. sculptum**	19	29	*T. terrestris*	Teodoro Sampaio	São Paulo	22°31’S 52°10’W	July 2008	CNC 1148
*A. sculptum**	14	13	*T. pecari*	Teodoro Sampaio	São Paulo	22°31’S 52°10’W	June 2002	CNC 702
*A. sculptum**		2	*T. pecari*	Teodoro Sampaio	São Paulo	22°31’S 52°10’W	May 2004	CNC 850
*A. sculptum**	13	14	*T. pecari*	Teodoro Sampaio	São Paulo	22°31’S 52°10’W	January 2005	CNC 978
*A. sculptum**	1	3	free living	Teodoro Sampaio	São Paulo	22°31’S 52°10’W	April 2006	CNC 1202
*A. sculptum**	1	2	free living	Teodoro Sampaio	São Paulo	22°31’S 52°10’W	November 2006	CNC 1203
*A. sculptum**	32	21	free living	Teodoro Sampaio	São Paulo	22°31’S 52°10’W	October 2005	CNC 1206
*A. sculptum**	23	29	free living	Teodoro Sampaio	São Paulo	22°31’S 52°10’W	August 2005	CNC 1207
*A. sculptum**	6	5	free living	Teodoro Sampaio	São Paulo	22°31’S 52°10’W	August 2005	CNC 1208
*A. sculptum**	7	7	free living	Teodoro Sampaio	São Paulo	22°31’S 52°10’W	July 2006	CNC 1209
*A. sculptum**	15	21	free living	Teodoro Sampaio	São Paulo	22°31’S 52°10’W	October 2005	CNC 1210
*A. sculptum**	2	7	free living	Teodoro Sampaio	São Paulo	22°31’S 52°10’W	January 2006	CNC 1211
*A. sculptum**	15	7	free living	Teodoro Sampaio	São Paulo	22°31’S 52°10’W	May 2005	CNC 1212
*A. sculptum**	16	20	free living	Teodoro Sampaio	São Paulo	22°31’S 52°10’W	November 2006	CNC 1214

_A. sculptum_*		67	free living	Teodoro Sampaio	São Paulo	22°31’S 52°10’W	August 2005	CNC 1215
_A. sculptum_*		200	free living	Teodoro Sampaio	São Paulo	22°31’S 52°10’W	August 2005	CNC 1220
_A. sculptum_*		213	free living	Teodoro Sampaio	São Paulo	22°31’S 52°10’W	August 2005	CNC 1221
_A. sculptum_*	66	8	free living	Teodoro Sampaio	São Paulo	22°31’S 52°10’W	August 2005	CNC 1222
_A. sculptum_*		100	free living	Teodoro Sampaio	São Paulo	22°31’S 52°10’W	October 2005	CNC 1224
_A. sculptum_*		130	free living	Teodoro Sampaio	São Paulo	22°31’S 52°10’W	October 2005	CNC 1226
_A. sculptum_*		89	free living	Teodoro Sampaio	São Paulo	22°31’S 52°10’W	October 2005	CNC 1228
_A. sculptum_*		100	free living	Teodoro Sampaio	São Paulo	22°31’S 52°10’W	July 2005	CNC 1230
_A. sculptum_*		100	free living	Teodoro Sampaio	São Paulo	22°31’S 52°10’W	July 2005	CNC 1231
_A. sculptum_*		120	free living	Teodoro Sampaio	São Paulo	22°31’S 52°10’W	July 2005	CNC 1232
_A. sculptum_*	62	47	free living	Teodoro Sampaio	São Paulo	22°31’S 52°10’W	September 2005	CNC 1235
_A. sculptum_*	6	3	free living	Teodoro Sampaio	São Paulo	22°31’S 52°10’W	October 2005	CNC 1237
_A. sculptum_*	15	34	free living	Teodoro Sampaio	São Paulo	22°31’S 52°10’W	July 2006	CNC 1238
_A. sculptum_*	1	38	free living	Teodoro Sampaio	São Paulo	22°31’S 52°10’W	August 2005	CNC 1242
_A. sculptum_*	15	7	free living	Teodoro Sampaio	São Paulo	22°31’S 52°10’W	October 2005	CNC 1244
_A. sculptum_*	41	52	free living	Teodoro Sampaio	São Paulo	22°31’S 52°10’W	July 2005	CNC 1247
_A. sculptum_*	7	12	free living	Teodoro Sampaio	São Paulo	22°31’S 52°10’W	July 2006	CNC 1248
_A. sculptum_*	8	12	free living	Teodoro Sampaio	São Paulo	22°31’S 52°10’W	November 2006	CNC 1251
_A. sculptum_*		1	_C. familiaris_	Adrianópolis	Paraná	24°39’S 48°59’W	August 2012	CNC 2254
A. sculptum $^{+}$		1	_D. albiventris_	Teodoro Sampaio	São Paulo	22°31’S 52°10’W	June 2008	CNC 1134
A. sculptum $^{+}$*	14	27	_C. thous_	Barão de Melgaço	Mato Grosso	16°11’S 55°58’W	July 2004	CNC 868
A. sculptum $^{+}$*	18	30	_L. pardalis_	Chapada Gaúcha	Minas Gerais	15°18’S 45°37’W	July 2009	CNC 1452
A. cajennense s. l.	1		_H. hydrochaeris_	Marabá	Pará	5°22’S 49°7’W	July 2001	CNC 503
A. cajennense s. l.	1		_T. terrestris_	Governador Jorge Teixeira	Rondônia	10°31’S 62°38’W	December 2000	CNC 413
A. cajennense s. l.	3		free living	Governador Jorge Teixeira	Rondônia	10°31’S 62°38’W	June 2001	CNC 541
A. cajennense s. l.	3		free living	Governador Jorge Teixeira	Rondônia	10°31’S 62°38’W	December 2001	CNC 579
A. cajennense s. l.	2		free living	Governador Jorge Teixeira	Rondônia	10°31’S 62°38’W	February 2009	CNC 1409
A. cajennense s. l.	81		_M. tridactyla_	Araguaína	Tocantins	7°11’S 48°12’W	April 2013	CNC 2574
A. cajennense s. l.	1		free living	Araguaína	Tocantins	7°11’S 48°12’W	May 2013	CNC 2575
A. cajennense s. l.	2		_P. tajacu_	Araguatins	Tocantins	5°39’S 48°7’W	July 2012	CNC 2263
A. cajennense s. l.	1		_C. familiaris_	Caseara	Tocantins	9°16’S 49°57’W	May 2009	CNC 1710
A. cajennense s. l.	6		_E. caballus_	Goiatins	Tocantins	7°42’S 47°18’W	September 2007	CNC 1059
A. cajennense s. l.	3		_B. taurus_	Lagoa da Confusão	Tocantins	10°47’S 49°37’W	August 2009	CNC 1795
A. cajennense s. l.	36		_M. tridactyla_	Lagoa da Confusão	Tocantins	10°47’S 49°37’W	May 2013	CNC 2573
A. cajennense s. l.	1		_T. tetradactyla_	Presidente Kennedy	Tocantins	8°32’S 48°30’W	December 2012	CNC 2330
A. cajennense s. l.	1		free living	Ipiaú	Bahia	14°8’S 39°44’W	September 2011	CNC 2628
A. cajennense s. l.	1		_E. caballus_	Gravatá	Pernambuco	8°12’S 35°33’W	January 2008	CNC 1324
A. cajennense s. l.	1		_E. caballus_	Gravatá	Pernambuco	8°12’S 35°33’W	December 2007	CNC 1327
A. cajennense s. l.	1		_E. caballus_	Sairé	Pernambuco	8°19’S 35°42’W	January 2008	CNC 1322

A. cajennense s. l.	1		*H. sapiens*	Brasília	Distrito Federal	15°47’S 47°52’W	January 1999	CNC 195
A. cajennense s. l.	2		*P. onca*	Brasília	Distrito Federal	15°47’S 47°52’W	January 1999	CNC 192
A. cajennense s. l.	1		*C. brachyurus*	Mineiros	Goiás	17°34’S 52°33’W	May 2005	CNC 1684
A. cajennense s. l.	1		*P. onca*	Mineiros	Goiás	17°34’S 52°33’W	August 2004	CNC 1658
A. cajennense s. l.	1		*S. venaticus*	Mineiros	Goiás	17°34’S 52°33’W	November 2002	CNC 819
A. cajennense s. l.	2		*B. taurus*	Poconé	Mato Grosso	16°15’S 56°37’W	August 1997	CNC 93
A. cajennense s. l.	1		*C. familiaris*	Sinop	Mato Grosso	11°50’S 55°38’W	June 2012	CNC 2235
A. cajennense s. l.	3		*H. sapiens*	Sinop	Mato Grosso	11°50’S 55°38’W	June 2010	CNC 1648
A. cajennense s. l.	3		*P. tajacu*	Anaurilândia	Mato Grosso do Sul	22°11’S 52°43’W	March 2001	CNC 447
A. cajennense s. l.	2		*P. concolor*	Anaurilândia	Mato Grosso do Sul	22°11’S 52°43’W	November 2002	CNC 834
A. cajennense s. l.	1		*M. tridactyla*	Brasilândia	Mato Grosso do Sul	21°15’S 52°2’W	April 2000	CNC 292
A. cajennense s. l.	7		*M. tridactyla*	Brasilândia	Mato Grosso do Sul	21°15’S 52°2’W	July 2000	CNC 333
A. cajennense s. l.	1		*M. tridactyla*	Brasilândia	Mato Grosso do Sul	21°15’S 52°2’W	August 2000	CNC 340
A. cajennense s. l.	1		*T. terrestris*	Bonito	Mato Grosso do Sul	21°7’S 56°28’W	March 2002	CNC 624
A. cajennense s. l.	2		*H. hydrochaeris*	Corumbá	Mato Grosso do Sul	19°0’S 57°39’W	September 1997	CNC 101
A. cajennense s. l.	1		*H. hydrochaeris*	Corumbá	Mato Grosso do Sul	19°0’S 57°39’W	September 1997	CNC 102
A. cajennense s. l.	2		*T. pecari*	Corumbá	Mato Grosso do Sul	19°0’S 57°39’W	September 1997	CNC 95
A. cajennense s. l.	3		free living	Corumbá	Mato Grosso do Sul	19°0’S 57°39’W	September 2008	CNC 1158
A. cajennense s. l.	1		*C. familiaris*	Miranda	Mato Grosso do Sul	20°14’S 56°22’W	November 2008	CNC 1709
A. cajennense s. l.	1		*P. concolor*	Naviraí	Mato Grosso do Sul	23°3’S 54°11’W	May 2004	CNC 950
A. cajennense s. l.	1		*M. tridactyla*	Nova Alvorada do Sul	Mato Grosso do Sul	21°27’S 54°23’W	April 2005	CNC 896
A. cajennense s. l.	4		*M. tridactyla*	Santa Rita do Pardo	Mato Grosso do Sul	21°18’S 52°49’W	August 2000	CNC 343
A. cajennense s. l.	1		*M. tridactyla*	Santa Rita do Pardo	Mato Grosso do Sul	21°18’S 52°49’W	August 2000	CNC 346
A. cajennense s. l.	1		free living	Chapada Gaúcha	Minas Gerais	15°18’S 45°37’W	May 2012	CNC 2178
A. cajennense s. l.	1		*H. sapiens*	Marliéria	Minas Gerais	19°42’S 42°43’W	October 2012	CNC 2370
A. cajennense s. l.	1		free living	Marliéria	Minas Gerais	19°42’S 42°43’W	October 2013	CNC 2533
A. cajennense s. l.	1		*E. caballus*	Uberlândia	Minas Gerais	18°55’S 48°16’W	September 2010	CNC 1669
A. cajennense s. l.	1		free living	Araçariguama	São Paulo	23°26’S 47°3’W	March 1998	CNC 127
A. cajennense s. l.	3		*M. tridactyla*	Araçoiaba da Serra	São Paulo	23°30’S 47°36’W	November 2010	CNC 1748
A. cajennense s. l.	1		*C. brachyurus*	Franca	São Paulo	20°32’S 47°24’W	October 2001	CNC 518
A. cajennense s. l.	1		*E. caballus*	Guaraci	São Paulo	20°29’S 48°56’W	January 2005	CNC 911
A. cajennense s. l.	1		*H. sapiens*	Pauliceia	São Paulo	21°19’S 51°49’W	April 2001	CNC 452
A. cajennense s. l.	1		*L. tigrinus*	Pedreira	São Paulo	22°44’S 46°54’W	January 1999	CNC 207
A. cajennense s. l.	1		*D. albiventris*	Presidente Epitácio	São Paulo	21°45’S 52°6’W	April 2002	CNC 655
A. cajennense s. l.	1		free living	Ribeirão Grande	São Paulo	24°5’S 48°21’W	July 2005	CNC 959
A. cajennense s. l.	1		free living	Santa Cruz do Rio Pardo	São Paulo	22°53’S 49°37’W	April 2007	CNC 1019
A. cajennense s. l.	1		*C. familiaris*	São Paulo	São Paulo	23°32’S 46°38’W	November 2000	CNC 367
A. cajennense s. l.	1		*H. sapiens*	Sorocaba	São Paulo	23°30’S 47°27’W	April 2013	CNC 2454
A. cajennense s. l.	1		*S. villosus*	Sorocaba	São Paulo	23°30’S 47°27’W	October 2007	CNC 1078

A. cajennense s. l.	1		*P. tajacu*	Teodoro Sampaio	São Paulo	22°31'S 52°10'W	April 2002	CNC 654
A. cajennense s. l.	1		*P. tajacu*	Teodoro Sampaio	São Paulo	22°31'S 52°10'W	July 2001	CNC 1030
A. cajennense s. l.	1		*T. pecari*	Teodoro Sampaio	São Paulo	22°31'S 52°10'W	May 2002	CNC 682
A. cajennense s. l.	1		free living	Teodoro Sampaio	São Paulo	22°31'S 52°10'W	July 2005	CNC 1246
A. cajennense s. l.	1		*E. caballus*	Rosário do Ivaí	Paraná	24°16'S 51°16'W	February 2001	CNC 441
A. cajennense s. l.	1		*H. sapiens*	Angatuba	São Paulo	23°29'S 48°24'W	October 2010	CNC 1742
*A. cajennense s. l.**	10		*C. familiaris*	Cristalândia	Tocantins	10°36'S 49°11'W	August 2011	CNC 2619
*A. cajennense s. l.**	2		*L. pardalis*	Barão de Melgaço	Mato Grosso	16°11'S 55°58'W	September 2001	CNC 801
*A. cajennense s. l.**	1		*H. sapiens*	Jauru	Mato Grosso	15°20'S 58°51'W	September 2002	CNC 733
*A. cajennense s. l.**	1		*T. tetradactyla*	Brasilândia	Mato Grosso do Sul	21°15'S 52°2'W	July 2000	CNC 334
*A. cajennense s. l.**	1		*H. sapiens*	São Roque de Minas	Minas Gerais	20°14'S 46°21'W	April 2007	CNC 1024
*A. cajennense s. l.**	1		*H. sapiens*	Americana	São Paulo	22°44'S 47°19'W	September 2011	CNC 2049
*A. cajennense s. l.**	1		*H. sapiens*	Atibaia	São Paulo	23°7'S 46°33'W	September 2000	CNC 371
*A. cajennense s. l.**	1		*H. sapiens*	Campinas	São Paulo	22°54'S 47°3'W	September 2002	CNC 748
*A. cajennense s. l.**	1		*Tyto alba*	Guarulhos	São Paulo	23°27'S 46°31'W	August 2003	CNC 797
*A. cajennense s. l.**	1		*C. familiaris*	Indaiatuba	São Paulo	23°5'S 47°13'W	August 2000	CNC 369
*A. cajennense s. l.**	2		*Capra hircus*	São Paulo	São Paulo	23°32'S 46°38'W	June 2002	CNC 715
*A. cajennense s. l.**	1		*H. sapiens*	São Paulo	São Paulo	23°32'S 46°38'W	July 2000	CNC 372
*A. cajennense s. l.**	1		*D. azarae*	Teodoro Sampaio	São Paulo	22°31'S 52°10'W	August 2009	CNC 1443
*A. cajennense s. l.**	1		*H. sapiens*	Teodoro Sampaio	São Paulo	22°31'S 52°10'W	December 2000	CNC 611
*A. cajennense s. l.**	2		free living	Teodoro Sampaio	São Paulo	22°31'S 52°10'W	July 2006	CNC 1201
*A. cajennense s. l.**	48		free living	Teodoro Sampaio	São Paulo	22°31'S 52°10'W	August 2005	CNC 1216
*A. cajennense s. l.**	200		free living	Teodoro Sampaio	São Paulo	22°31'S 52°10'W	August 2005	CNC 1218
*A. cajennense s. l.**	222		free living	Teodoro Sampaio	São Paulo	22°31'S 52°10'W	August 2005	CNC 1219
*A. cajennense s. l.**	93		free living	Teodoro Sampaio	São Paulo	22°31'S 52°10'W	October 2005	CNC 1223
*A. cajennense s. l.**	124		free living	Teodoro Sampaio	São Paulo	22°31'S 52°10'W	October 2005	CNC 1225
*A. cajennense s. l.**	83		free living	Teodoro Sampaio	São Paulo	22°31'S 52°10'W	October 2005	CNC 1227
*A. cajennense s. l.**	150		free living	Teodoro Sampaio	São Paulo	22°31'S 52°10'W	July 2005	CNC 1233
*A. cajennense s. l.**	136		free living	Teodoro Sampaio	São Paulo	22°31'S 52°10'W	July 2005	CNC 1234
*A. cajennense s. l.**	1		free living	Teodoro Sampaio	São Paulo	22°31'S 52°10'W	June 2005	CNC 1240
*A. cajennense s. l.**	5		free living	Teodoro Sampaio	São Paulo	22°31'S 52°10'W	January 2006	CNC 1241
*A. cajennense s. l.**	1		*P. cancrivorus*	Jundiaí	São Paulo	23°11'S 46°53'W	October 1998	CNC 169

** Technically, all males were morphologically identified as *Amblyomma cajennense s.l.* However, by convenience only in this Table, males from lots containing females were assigned to the same species of the accompanying females. If a lot contained only male(s), it was assigned as *A. cajennense s.l.*

* TICKS COLLECTED AS NYMPHS

⁺ TICKS COLLECTED AS LARVAE
